# Butyl-Methyl-Pyridinium Tetrafluoroborate Confined in Mesoporous Silica Xerogels: Thermal Behaviour and Matrix-Template Interaction

**DOI:** 10.3390/ma14174918

**Published:** 2021-08-29

**Authors:** Ana-Maria Putz, László Almásy, Zsolt Endre Horváth, László Trif

**Affiliations:** 1 “Coriolan Drăgulescu” Institute of Chemistry, Bv. Mihai Viteazul, No. 24, 300223 Timisoara, Romania; 2 Institute for Energy Security and Environmental Safety, Centre for Energy Research, Konkoly-Thege Miklós út 29–33, 1121 Budapest, Hungary; 3 Institute for Technical Physics and Material Science, Centre for Energy Research, Konkoly-Thege Miklós út 29–33, 1121 Budapest, Hungary; horvath.zsolt.endre@energia.mta.hu; 4 Institute of Materials and Environmental Chemistry, Research Centre for Natural Sciences, Magyar Tudósok körútja 2, 1117 Budapest, Hungary; trif.laszlo@ttk.hu

**Keywords:** ionic liquid, mesoporous silica, thermal analysis, mass spectrometric evolved gas analysis, SANS

## Abstract

Organic-inorganic silica composites have been prepared via acid catalyzed sol-gel route using tetramethoxysilan (TMOS) and methyl-trimethoxysilane (MTMS) as silica precursors and *n*-butyl-3-methylpyridinium tetrafluoroborate ([bmPy][BF_4_]) as co-solvent and pore template, by varying the content of the ionic liquid (IL). Morphology of the xerogels prepared using the ionic liquid templating agent were investigated using scanning electron microscopy and small angle neutron scattering (SANS). Thermal analysis has been used in order to evaluate the thermal and structural stability of the materials, in both nitrogen and synthetic air atmosphere. In nitrogen atmosphere, the IL decomposition took place in one step starting above 150 °C and completed in the 150–460 °C temperature interval. In synthetic air atmosphere, the IL decomposition produced two-step mass loss, mainly in the 170–430 °C temperature interval. The decomposition mechanism of the IL inside the silica matrix was studied by mass spectrometric evolved gas analysis (MSEGA). The measurements showed that the degradation of the IL’s longer side chain (butyl) starts at low temperature (above 150 °C) through a C-N bond cleavage, initiated by the nucleophilic attack of a fluorine ion.

## 1. Introduction

Ionic liquids (ILs) are green and recyclable alternatives to traditional organic solvents which gain more and more attention in inorganic nanomaterials synthesis [1]. ILs have been used as reaction medium for inorganic materials, exploiting the pre-organised structure of the aqueous IL solutions as template in generating porous nanomaterials [2]. Concerning the mechanism of the sol-gel reaction in presence of IL and silica precursors, it has been shown that the addition of small amount of IL to aqueous mixtures of silica precursors slows down the polycondensation reaction and produces primary silica particles of larger sizes [3]. Composite materials based on silica and prepared by sol-gel route typically have heterogeneous structure on nanometer length scales, assessible for the small-angle scattering methods. Small-angle neutron and X-ray scattering (SANS and SAXS) has been used for elucidating the morphology of silica–IL composites prepared with short chain ionic liquids, and showed the effect of the ionic liquid as catalyst and as a pore template [4,5,6,7,8]. In contrast, use of long chain ionic liquids as pore templates leads to various ordered mesoporous silica structures, similar to those prepared with conventional cationic surfactants [9,10]. In the present study, we explored IL–silica composites prepared with high proportion of a compact, short alkyl chain IL added into the reaction mixture of silica precursors. The structural changes occurring on the nanometer length scale affect the materials’ properties such as porosity, and controlling them can improve the materials’ suitability for further applications.

Many physicochemical properties of ILs change upon confinement in silica nanopores, because of the interaction of the IL cations and anions with the pore walls [11,12]. Mesoporous silica monoliths were functionalized with 2-(4-pyridylethyl)-triethoxysilane and N,N-dimethyl-pyridine-4-yl-(3-triethoxysilyl-propyl)-ammonium iodide and then were filled with 1-ethyl-3-methyl imidazolium ionic liquid, allowing one to tune the properties of the resulting gel. Thermal analysis showed that the modification of the silica pore walls with organic groups strongly affects the phase behavior of the confined ionic liquids, leading to complete suppression of the glassy state of the imidazolium ionic liquid, compared to unmodified silica monoliths [11]. In contrast, modification with N,N-dimethyl-pyridine-4-yl-(3-triethoxysilyl-propyl)-ammonium iodide silane with varying degree of monolith functionalization led to the appearance and disappearance of a presumed additional phase of the ionic liquid [13]. DTG curves of pyridinium ILs in montmorillonite (a very soft phyllosilicate mineral) show two distinct maxima in the range 200–570 °C and this two-step decomposition suggests the presence of a small amount of ILs physisorbed on the surface and having the same temperature of decomposition as the pure ionic liquid. The chemisorbed IL fraction was responsible for the intense second exothermic peak below 570 °C [14].

In most examples from the literature, the temperature of decomposition of pure ionic liquids is higher than that of the confined ionic liquid. Two ionic liquids, namely 1-ethyl-3-methylimidazolium acetate and 1-ethyl-3-methylimidazolium methanesulfonate were impregnated in two silica supports [15]. The ionic liquids were found to self-organize on the silica surface, and when used in a sorption desalination process at low temperature of 60 °C, these composites show exceptionally high theoretical working capacities ranging from 1 to 1.7 g_water/_g_sorbent_. Experimental tests using a laboratory scale desalinator showed that 1-ethyl-3-methylimidazolium acetate on a silica support under real operating conditions can produce 25 kg_water_ kg_sorbent_^–1^ day^–1^, which is more than double of the best yield achieved with silica gel [15].

The power of thermal analysis of composite materials can be substantially enhanced by using analysis of the evolved gases during heating and decomposition [16,17]. Thermogravimetric analysis coupled with mass spectrometry (TA-MS) has been successfully applied for analysis of decomposition products upon heating ionic liquids [18,19,20]. However, no such investigations appear to exist for the thermal behavior of ionic liquid–silica composites. In the present study, we present TA-MS analysis of the thermal decomposition of *n*-butyl-3-methylpyridinium tetrafluoroborate ([bmPy][BF_4_]) incorporated in silica xerogel.

Different applications of the ionic liquids grafted on silica were recently presented in the literature, such as: ionic liquids grafted on silica for changing the charging behaviors of cations and anions in supercapacitors [21]; silica grafted imidazolium-based ionic liquids as efficient heterogeneous catalysts for chemical fixation of CO_2_ [22]; mesoporous silica-supported ionic liquids as catalysts [23]; silica microcapsules containing phosphonium ionic liquid, used as healing agents against microcracks propagating into the epoxy networks [24], or imidazolium based ionic liquids confined into mesoporous silica MCM-41 and SBA-15 for carbon dioxide capture [25]. Confinement in silica matrix provides new opportunities to use the nano-size ionic liquid compartment for catalysis [26]. In another study, supported imidazolium-based ionic liquid films were used as catalysts, and proved to be efficient for benzoin condensations [27].

Many applications of ionic liquid–inorganic matrix composites can be found in recent reviews [28,29,30,31]. Short alkyl chain ionic liquids can be used as structure modifiers of mesoporous silica. Functionalized imidazolium based ionic liquids, 1-chloro-(2-hydroxyethyl)-3-methyl imidazole, 1-bromo-ethylamine-3-methyl imidazolium and chlorinated 1-carboxyethyl-3-methyl imidazolium, were synthesized and used as silica supported imidazolium ionic liquids for Cr(VI), Re(VII), Ce(IV) adsorption [32]. Silica gel solid nanocomposite electrolytes were obtained with interfacial conductivity exceeding the bulk Li-ion conductivity of the ionic liquid electrolyte filler [33].

In the present study, we developed new materials using a short chain ionic liquid as templating agent. Electron microscopy and small-angle neutron scattering were employed to resolve the structure of the prepared materials on the micron and nanometer length scales. Thermogravimetric and differential scanning calorimetric analyses were used in order to characterize the thermal behavior and thermal stability of the materials. Mass spectrometric evolved gas analysis coupled with the thermal analysis revealed the decomposition mechanism of the IL inside the silica matrix.

## 2. Materials and Methods

### 2.1. Sample Preparation

**Figure 1:**
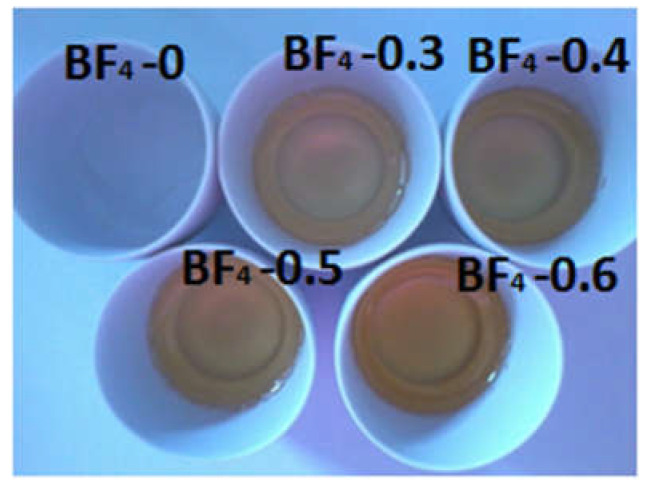
The obtained composite xerogels after gelation.

All reagents were used as received: N-butyl-3-methylpyridinium tetrafluoroborate ([bmPy][BF_4_]; for synthesis 98%, Merck (Darmstadt, Germany)); the silicon precursors: methyl-trimethoxysilane (MTMS, 98%, Merck) and tetramethoxysilane (TMOS, 98%, Merck); distilled water; and hydrochloric acid (HCl, 37%, S.C. Silal Trading SRL, Bucharest, Romania). Sols were prepared by mixing 0.0325 moles TMOS, 0.004 moles MTMS, 0.29 moles distilled water and 0.103 moles HCl acidic solution (pH = 2.8). The water and the HCl solution were added separately, dropwise during 20 min. The reaction mixture was mechanically vigorously stirred (30 min) in a round bottom flask until a homogenous sol was obtained. Next, ionic liquid solutions were prepared by dissolving different quantities of [bmPy][BF_4_] in 12.5 mL H_2_O, (Table 1) and added to the silica precursor mixture, with continuous stirring for 30 min. For comparison purposes, a blank sample was prepared using the same procedure, but without any ionic liquid; instead an equivalent quantity of water (12.5 mL) was added. The obtained sols were left to gel at room temperature. The IL containing mixtures gelled for the next day, in less than 24 h, whereas the blank sample gelled within 48 h. Shrinkage and syneresis are reduced during gelation and aging due to the adding of the ionic liquid into the synthesis mixture [8]. The formed gels were transparent monoliths without cracks. The xerogel of the blank sample was white, and with the increase of the IL amount, the pale yellow color of the samples became darker (Figure 1). These materials were crushed and grinded to powder form for further analyses, and were dried for 12 h at 100 °C.

### 2.2. Characterization Methods

FT-IR spectra were recorded in KBr pellets using a JASCO FT/IR-4200 apparatus (SpectraLab, Shimadzu, Kyoto, Japan). Samples for transmittance measurements were prepared typically by mixing 3 mg of sample with 600 mg of KCl. The powdered samples were pelletized by applying 7.5 tons for several minutes under vacuum of several mm Hg.

Scanning electron microscopy (SEM) analysis was performed using an FEI Scios DualBeam System (Thermo Scientific, Waltham, MA, USA) equipped with an X-Max^N^ 20 SDD energy dispersive spectroscopy (EDS) detector (Oxford Instruments, Abingdon, UK). During EDS analysis an accelerating voltage of 5 keV was used.

Small-angle neutron scattering measurements were performed on the *Yellow Submarine* instrument located at BNC in Budapest (Hungary) [34]. A collimation distance of 5 m and circular beam apertures of 25 and 8 mm in diameter defined the incoming beam divergence, while sample to detector distances of 1.2 and 5.5 m and neutron wavelengths of 0.4 nm and 1.0 nm defined the accessible momentum transfer range of 0.06–3.8 nm^−1^. The scattered neutrons were detected by a two-dimensional position-sensitive BF_3_ gas detector (64 × 64 cells of 1 cm × 1 cm). The raw data were corrected for sample transmissions and background scattering of the empty cell, and converted to absolute units by comparison with the incoherent scattering of a 1 mm thick water sample. The powdered samples were filled into Hellma quartz cells of 2 mm flight path, and the measurements were done at room temperature.

Nitrogen sorption was measured at 77K with a QuantaChrome Nova 1200e analyser (Quantachrome Instruments, Boynton Beach, FL, USA). Before measurements, each sample was outgassed at 100 °C for several hours. The NovaWin software was used to evaluate the isotherms. Specific surface area was determined by Brunauer–Emmett–Teller (BET) method in the relative pressure (P/P_0_) range 0.01–0.25. The total pore volumes were determined using the data at P/P_0_ closest to 1. Pore diameter and pore size distributions were evaluated by density functional theory (DFT) method.

Two series of thermal measurements were performed on a Setaram Labsys Evo (Lyon, France) TG-DSC system, in flowing high purity nitrogen (99.999%; flow rate 60 mL/min) and separately, in synthetic air (20% O_2_ + 80% N_2_; flow rate 80 mL/min) atmosphere. Samples were weighed into 100 μL platinum crucibles (the reference cell was empty) and where heated from 25 °C to 700 °C with a heating rate of 10 °C/min. The measurements were performed as follows: first a set of three blanks were recorded during the night (to obtain the most stable baselines), with the same parameters as the subsequent sample measurements. The sample measurements were performed during the day. The obtained data were blank corrected and further processed with the thermoanalyzer’s software (Calisto Processing, AKTS, Sierre, Switzerland). Mass losses are determined by the horizontal calculation method. In the case of DTG curves, the peak maximum (minimum) temperature is given, while in the case of heat flow (DSC) curves, both values the peak maximum and the onset temparature values are given. Enthalpies are calculated from the integrated peak areas, by the tangential sigmoid baseline calculation method, considering the mass at the first integration limit. The thermal analyzer (both the temperature scale and calorimetric sensitivity) was calibrated by a multipoint calibration method, in which seven different certified reference materials were used to cover the thermal analyzer’s entire operating temperature range. In parallel with the thermal measurements, the analysis of evolved gases and volatiles was performed on a Pfeiffer Vacuum Omni Star™ (Asslar, Germany) mass spectrometric evolved gas analysis system (MSEGA), which was connected to the above mentioned thermal analyzer. MSEGA measurements were performed in flowing (90 mL/min) high purity (99.9999%) helium atmosphere, with a heating rate of 20 °C/min. The gas splitter and transfer line to the mass spectrometer was thermostated to 260 °C, while the inlet was heated to 120 °C. The measurements were done in SEM Bargraph Cycles acquisition mode, where the *m/z* interval of 5–155 was continuously scanned with a speed of 50 ms/amu. The spectrometer was operated in electron impact mode. Instrumental parameters are collected in the Supplementary Information.

## 3. Results

### 3.1. FT-IR Spectroscopy

**Figure 2:**
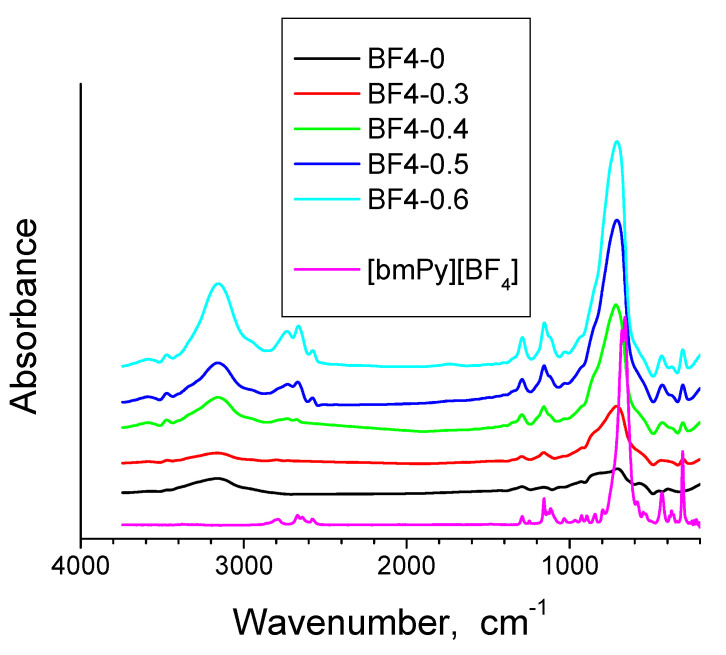
FTIR spectra for ionic liquid [bmPy][BF_4_] and the IL containing silica xerogels.

**Figure 3:**
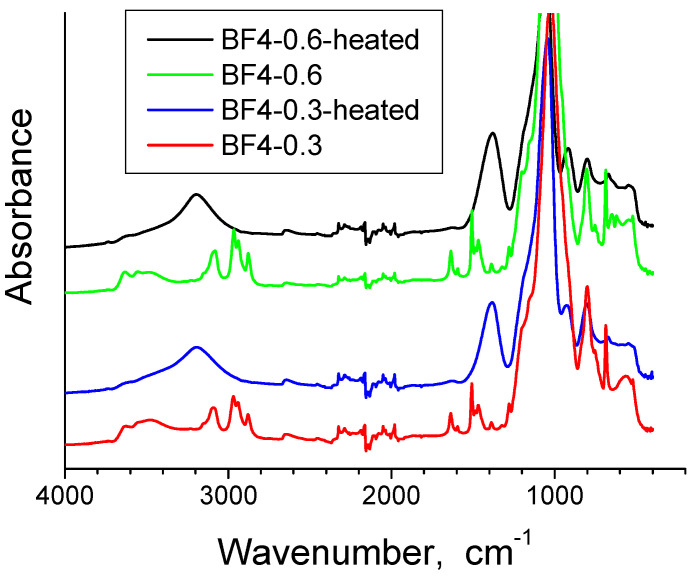
FTIR spectra for samples BF4-0.3 and BF4-0.6 at room temperature and after thermal treatment at 700 °C in air.

The FT-IR spectra for all xerogel samples and the ionic liquid are presented in Figure 2 and the band assignments are given in Table 2. Two xerogel samples have been selected for further IR analysis, the one with the lowest IL content, BF4-0.3 and the one with the highest IL content, BF4-0.6, in the xerogel state and after thermal treatment at 700 °C in air for one hour (Figure 3) The characteristic IL absorption bands of the ionic liquid in the 900–1100 cm^−1^ region overlay with the absorption bands of the silica. With increase of the IL content in silica, the IL bands become gradually stronger (Figure 2 and Table 2).

The band at 3553 cm^−1^ for BF4-0.6 xerogel sample (respectively 3479 cm^−1^ for BF4-0.3 xerogel sample), corresponds to the overlapping of the O-H stretching bands of hydrogen-bonded water molecules (H-O-H... H) and SiO-H stretching of surface silanols hydrogen-bonded to molecular water (SiO-H…H_2_O) [35]. The band between 3081–3194 cm^−1^ is due to the OH stretching vibrations [36]. This band changes with thermal treatment and is intense in xerogels and it decreases in the heated samples, but does not disappear completely. The adsorbed water deformation vibration band appears at 1636–1637 cm^−1^ only in xerogel samples [35,36,37]. In the 1981–2324 cm^−1^ range, the bands from the four analysed samples contain vibrations of organic residue, molecular water and silica network [38]. The intense and broad band between 1057 and 1024 cm^−1^ is assigned to the Si-O-Si asymmetric stretching vibrations [35], and is shifted to higher wave numbers after calcination, due to the strengthening of the silica matrix [39] and elimination of the IL. The symmetric stretching vibrations of Si-O-Si appear at 800 cm^−1^ and 680 cm^−1^ [35,40]. The band at 1635 cm^−1^ is assigned also to the SiO_2_ network vibration bands (often overlapped by molecular water band) [38]. The stretching vibrations of alkyl chains appear at 2965–2977 cm^−1^ and at 2878–2879 cm^−1^ [41]; these bands are present in the xerogel samples and disappear after thermal treatment, demonstrating therefore that the alkyl chains of the ionic liquid were removed. At 1277–1279 cm^−^^1^, the symmetric CH_3_ bending band from Si–CH_3_ [37,42] is observed only in xerogel samples. For the IL containing silica, the characteristic band of BF_4_ group at 765 cm^−1^ [43] overlaps with the symmetric stretching vibrations of Si-O-Si appearing at 800 cm^−1^ and 680 cm^−1^ [44]. The band around 687 cm^−1^ can also be assigned to the symmetric stretching vibration of the C-Si bond (in literature this assignment is made at 676 cm^−1^) [45], but at 700 °C, this band almost disappears since the CH_3_ groups from the silica matrix were also eliminated. The band at 1468 cm^−1^ is present only in the xerogel samples and it can be assigned to the C-H bond stretching vibrations of the alkyl chain [43]. The bands at 1468 cm^−1^ and 1507 cm^−1^ are also due to the pyridinium group and are present in the bulk IL and in the xerogel samples as well [46]. We suppose that in the thermally treated samples, the bands from 1508 cm^−1^ and 1468 cm^−1^ are shifted to 1383 cm^−1^. The band at 1383 cm^−1^ is present also in the bulk IL and is generally assigned to stretching vibrations of C-H bond [41] and C-C bond [47]. This shows that some IL still remains entrapped in the silica matrix, after the one hour thermal treatment, while the entrapped IL is also affecting the changes in the silica network vibrations.

### 3.2. Electron Microscopy and EDS Analysis

**Figure 4:**
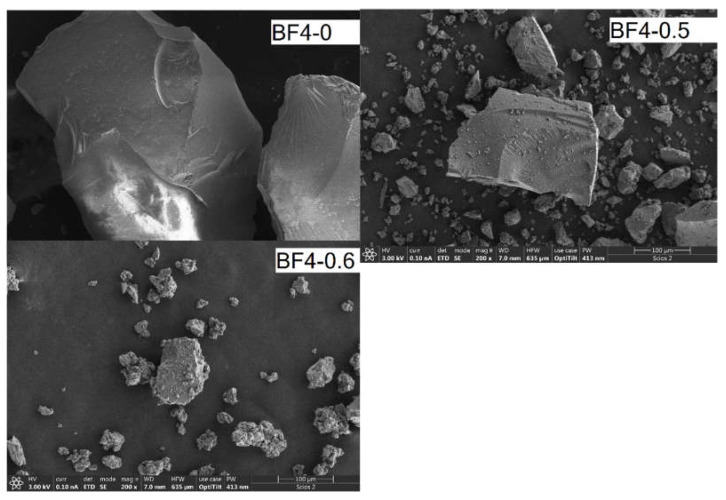
Characteristic SEM images of IL-silica composite samples. The scale bar corresponds to 100 µm.

Morphology of the materials has been characterized by scanning electron microscopy (Figure 4). EDS spectra were taken from several selected areas of each sample (Figure S1 in the Supplementary Materials). The particles obtained after grinding have irregular shapes typical for xerogels; the characteristic images are shown in Figure 4. Higher magnification SEM images are shown in Figure S2. A noticeable difference between the materials can be seen in the particle surfaces, which appear smooth in the materials prepared without, or with a low amount of ionic liquid. The fractured particles in sample BF4-0.6, prepared with the highest amount of ionic liquid show rough surfaces. EDS analysis of selected areas of the micron sized particle surfaces confirmed the gradual change of the ionic liquid content in the solid composite samples (Table S1).

### 3.3. Nitrogen Porosimetry

**Figure 5:**
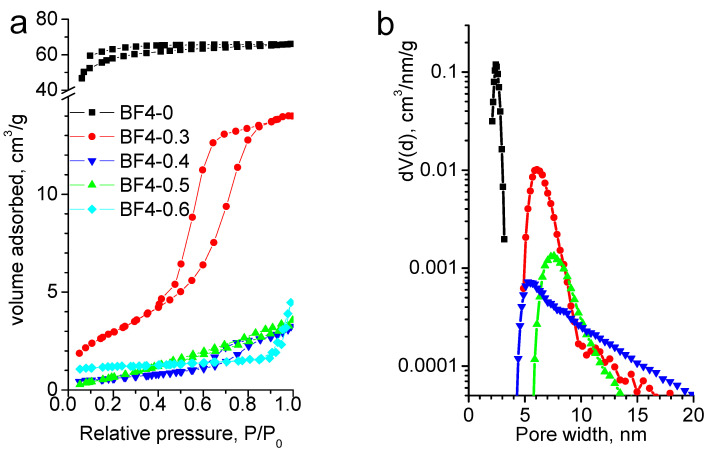
Nitrogen adsorption-desorption isotherms (**a**) and pore size distribution (**b**) for IL-silica composite samples.

The nitrogen sorption isotherms are shown in Figure 5a, and the textural parameters are collected in Table 3. The blank and BF4-03 samples present a type IV isotherm with H2 hysteresis type, specific for complex pore structure. The other three samples show type II isotherms, characteristic for nonporous materials. Sample BF4-0.5 presents H3 type hysteresis.

The specific surface areas for all composite materials are rather small. With the increase of IL concentration the total pore volume decreases. The pore diameters first increase, then decrease with increasing IL content (Table 3). The narrow pore size distribution in the BF4-0 and BF4-0.3 samples shifts to the larger values for samples BF4-0.4 and BF4-0.5, possibly due to the dominance of the intergrain surfaces (Figure 5b).

### 3.4. Thermal Analysis

**Figure 6:**
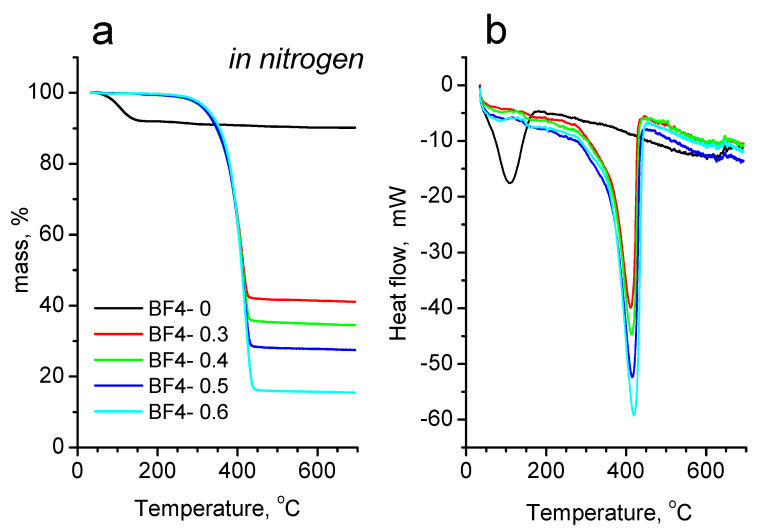
Mass loss (TG) (**a**) and heat flow (DSC) (**b**) curves in inert atmosphere.

**Figure 7:**
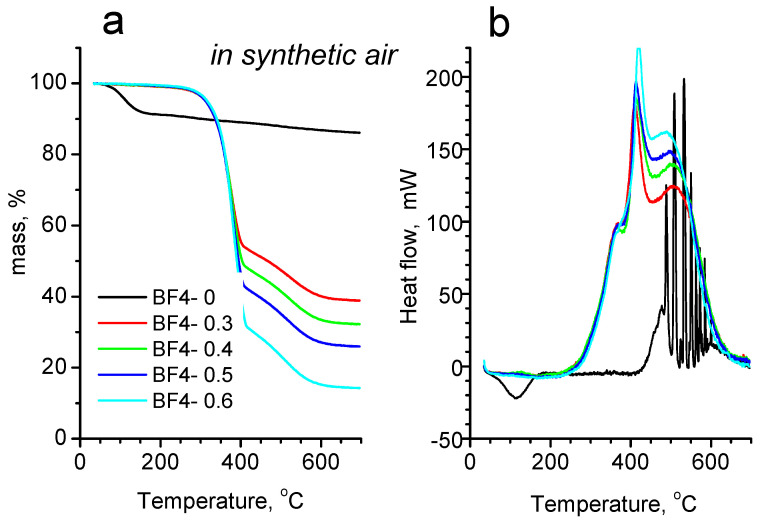
Mass loss (TG) (**a**) and heat flow (DSC) (**b**) curves in air atmosphere.

The results of the thermal investigations in inert and oxidative atmosphere are shown in Figure 6 and Figure 7, while the mass losses at characteristic temperature intervals are also presented in Table S2. In inert atmosphere (Figure 6), all samples show a single step mass loss. On the TG trace of the IL–free sample BF4-0, there is a small mass loss of 8% between 40 and 170 °C, accompanied by a small endotherm on the corresponding heat flow curve, which is the result of the evaporation of physically bound water. From 170 °C up to 460 °C, there is another small mass loss (Δm = 1.38%), which is due to the release of water from the hydroxyl groups in the particles. The IL containing samples start to degrade above 170 °C, and up to this temperature practically there is no significant mass loss (around 0.3%). Between 170 °C and 460 °C there is a single mass loss step, accompanied by a sharp endotherm (Figure 6b). In this step, the complex pyrolytic degradation of the IL takes place. On the comparative graph (Figure 6a) it can be seen, that the amount of the mass loss increases obviously with the increasing IL content, and the final temperature of mass loss process shifts towards higher temperatures by about 4–5 °C for the subsequent samples (BF4-0.3: 435.4 °C; BF4-0.4: 439.5 °C; BF4-0.5: 444.3 °C and BF4-0.6: 449.2 °C). Above 460 °C up to the end of the measurement, only a slight mass loss occurs, which can be attributed to the slow oxidation of the remaining carbon by the residual oxygen content of the carrier gas and the oxygens from the Si-O framework. The solid residues after the measurement were black colored, which means that there is carbon left from the pyrolysis of the organic part of IL.

Comparing the thermal degradation in inert gas and in synthetic air, a characteristic difference can be seen on both the mass loss curves and the heat flow curves, too. In synthetic air (Figure 7), the IL-free BF4-0 sample shows a single mass loss step between 40 and 170 °C, with a very similar amount of mass loss (Δm = –8.39%) like in the case of heating in nitrogen atmosphere. From 170 °C up to the end of the heating an additional 5.41% of mass is lost. The higher total mass loss value compared to the corresponding mass loss in inert atmosphere could have two origins: a higher dehydroxylation process in air, or could also be the slow oxidative burning of the small amount of organics of the gel precursors entrapped into the silica framework. The latter is supported by the direction of the heat flow curve, which turns exothermic above 400 °C. The sharp successive exothermic peaks could be the results of internal relaxation processes.

Examining the mass loss curves of the IL containing samples in synthetic air, a two-step mass loss profile can be seen on each curve, accompanied by three overlapped exothermic peaks on the corresponding heat flow curves. From the starting of the measurements up to 170 °C, between 0.2 and 0.8% of mass is lost, which is the result of the evaporation of moisture. Surprisingly, in air between 170 and 430 °C a larger, while from 430 °C up to the end of the measurement a smaller, but broader mass loss step can be seen. After the heat treatments in synthetic air, the materials became light brownish (or almost white), meaning that most of the organic content was burnt out, with some small residue remaining only. If the values of the mass losses on the whole measurement temperature interval are compared for the measurements performed in nitrogen and synthetic air, only a slight difference (mass loss difference between 1.07 and 3.12%) can be seen in the favor of the measurements performed in air (Table S2). These values correspond to the small amount of carbon remained in the sample during the measurements under pyrolytic conditions. The reason of the two-step mass loss of IL containing samples in air is still under investigation, we hypothesize that because of the presence of oxygen, some more stable, partly oxidized, higher molecular weight products are forming inside the pores of the silica matrix, which hinder the evaporation and burning of the organic products. At higher temperatures, above 430 °C, these partly oxidized fragments break up and leave the silica framework by slow diffusion and burning.

### 3.5. Mass Spectroscopic Analysis of the Evolved Gases

In order to investigate the thermal decomposition mechanism of the IL inside the silica matrix under inert atmosphere, measurements were repeated, where two samples (BF4-0 and BF4-0.4) in parallel with the thermal measurements were investigated by mass spectrometric evolved gas analysis, too. In Figure S3, the ion current curves of water (*m/z*–18 molecular ion, *m/z*–17 hydroxyl ion) are plotted against temperature. It can be seen, that the mass loss observed at the start of heating is indeed caused by the loss of the physisorbed water (well defined peaks on *m/z*–18 and –17 between 35 and 175 °C; peak maximum at 95 °C). Between 225 and 375 °C another small and broad peak can be seen on the *m/z*–18 and –17 curves, which corresponds to the loss of water due to dehydroxylation of the silica framework surface.

In Figure S4 and Figure S5, the ion current curves of characteristic fragments to the hydrocarbon side chains and pyridyl group are plotted against temperature. It can be seen, that in accordance with the literature data [19], the degradation of the IL’s longer side chain (butyl) starts at relatively low temperature (above 150 °C) through a C-N bond cleavage, initiated by the nucleophilic attack of a fluorine ion. Fragments with higher masses correspond to some fluorine containing hydrocarbons (*m/z*–100, 85, 81 and 66). The parallel rundown of *m/z*–100 and *m/z*–81 curves clearly demonstrates their relation and loss of a fluorine atom. The lower *m/z*-s are characteristic fragments of the fluorine containing hydrocarbon chain: *m/z*–56 (butane-C_4_H_8_) formed from the direct C-N bond cleavage and one hydrogen loss; *m/z*–33 (CH_2_F); *m/z*–47 (CH_2_CH_2_F).

The degradation of the alkyl chain on the pyridyl group at lower temperatures takes place only partly, because at higher temperatures (above 275 °C), intact, non-degraded [bmPy] cation can also be detected (*m/z*–149, [bmPy]-H molecular ion). The *m/z*–134 corresponds to {[bmPy]-H}-CH_3_, while *m/z*–107 corresponds to [bmPy]-C_3_H_7_ (a propyl group is lost from the [bmPy]’s butyl chain. The *m/z*–93 peak is the methyl pyridinium ion {[bmPy]-C_4_H_9_}. Species with masses below *m/z*–92 are most probably fragmentation products of the [bmPy] cation. The evaporation of BF_4_^-^ anion can be detected above 280 °C (*m/z*–68, not shown).

Traces of water evolve in two steps (Figure S3), between 35 and 125 °C the loosely bound moisture (not detected by the TG), while between 125 and 240 °C the more strongly bound water is released (around 0.42%). Formation of water together with CO_2_ can be detected at temperatures between 275 and 460 °C, where the water possibly results from the reaction of the hydrogens of [bmPy] with the hydroxyls and oxygens from the silica structure, while the source of the CO_2_ is also the reaction of carbon resulted from the degradation of the [bmPy] with the oxygens from the silica framework. The analog mass spectra of the volatiles evolved at 335.4 °C is shown in Figure S5, while spectra of the volatiles released at 415 °C can be seen in Figure S6.

**Figure 8:**
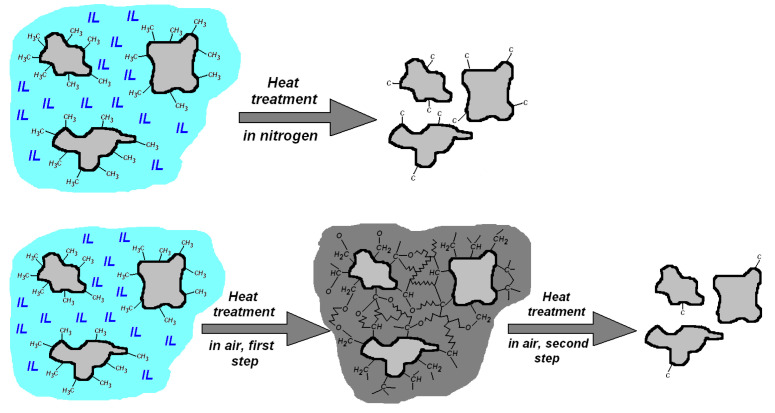
Schematic view of the structural changes occurring as one-step process during heating in nitrogen and in two steps when heating in synthetic air. Color codes: light grey–silica; light blue–ionic liquid, dark grey–partly oxidized organic moieties from the IL.

The main steps of the morphological changes and evaporation and burning of the organic components during heat treatment in inert and oxidizing atmospheres are depicted in Figure 8. In nitrogen atmosphere, the heating results in mass loss of most of the organic component, with some carbon remaining that gives a characteristic black color. Shrinkage of the silica grains occurs due to collapse of the pores filled with IL. When heating in air, the first mass loss step ends with forming a two-phase structure consisting of compacted silica grains mixed with partly oxidized organic remnants of the IL, which oxidize upon further heating and leave in the second mass loss step.

### 3.6. SANS Analysis of the Particle and Agglomerate Morphology

**Figure 9:**
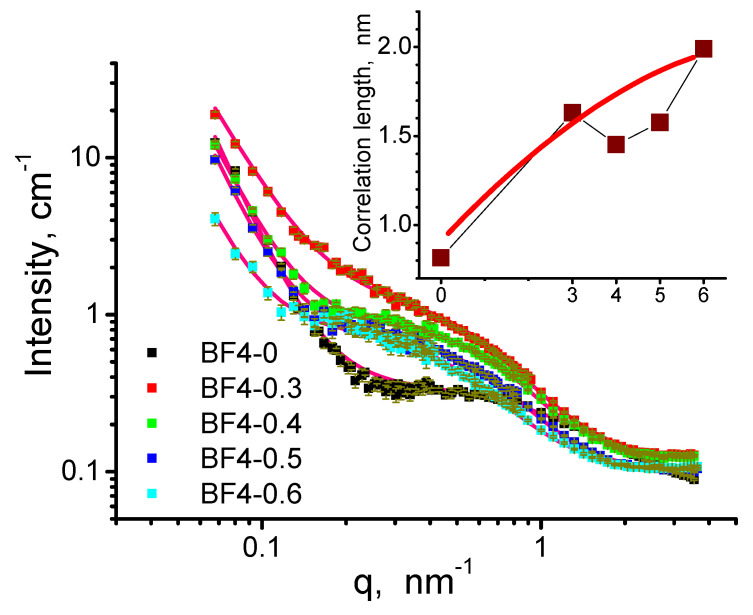
Small angle scattering patterns of the silica–IL composites. The red solid lines are the model fits to Equation (1). In the inset, the values of the correlation length *ξ* are shown for the different samples. The solid red line indicates the trend.

The SANS curves of the nanocomposites are shown in Figure 9. The scattering data show the characteristic features of dry mesoporous xerogels, which usually display a heterogeneous structure with two or more distinct levels of characteristic length scales [48,49,50,51,52,53,54]. The broad plateau or hump in the momentum transfer (*q*) range 0.1–1 nm^−1^ indicates heterogeneities of a few nanometers in size, which are usually assigned to the primary silica particles that form in the sol-gel process [4,48,49,50]. The sharp increase of the scattering intensity towards low *q*, below *q* = 0.1 nm^−1^ corresponds to size range above 100 nm, and is due to the silica–air, and the silica–IL interfaces in the macroscopic porous structure of the dry powdered material. The neutron scattering length densities of the IL molecules, due to the presence of 16 H atoms, is much closer to that of air, than of bulk SiO_2_. Therefore, the SANS experiment is most sensitive to the structure of the silica xerogel.

The whole scattering pattern was fitted by empirical Equation (1), which combines the two structural motives:

(1) $$I\left(q\right)=A\;q^{-p}+\frac{B}{\left(1+q^{2}\xi^{2}\right)}+Bg\;$$

where *A* and *B* are scaling factors for the two terms, *p* is the power exponent describing the interface of the agglomerated gel particles, *ξ* is the correlation length from the second term, which indicates the characteristic size of the primary silica particles, and *Bg* is an adjustable flat background term, which accounts for the isotropic, *q*-independent contribution to the scattering intensity mainly due to the hydrogen content of the materials. The model describes reasonably well the measured scattering intensities. The values of the *p* exponent are in the range of 3.6–4.1, indicating a more or less sharp surface of the gel particle agglomerates. The characteristic size of the smaller, primary silica gel particles displays an increasing trend with increasing amount of the embedded IL, as shown in the inset of Figure 9. This effect can be explained by the slowing of the condensation process in the presence of the non-reacting IL co-solvent in the reaction mixtures [3,4]. It should be noted, that there are several approximations used to model the high *q* region of silica xerogels, and all they employ a term containing a characteristic size parameter. In [3,55] the simplest model for compact particles was used, the Guinier term combined with a power law scattering for accounting for the particle aggregates. To account for the scattering arising from different structures of different sizes, these terms were combined by the empirical equation used by Beaucage [56]. Another frequently employed approximation is the correlation length model, followed by us and used also in [57]. Here the characteristic size appearing in the scattering curves can be interpreted as polydisperse particles as well as interparticle holes, or dense interconnected particle chains in which scattering from particles or holes cannot be distinguished solely based on the scattering pattern. Details of interpretation of scattering intensity data from fractal structures characteristic to silica aerogels can be found, e.g., in a seminal paper by Teixeira [58].

## 4. Discussion

Thermal behavior of neat IL usually consists of a one-step decomposition at high tempertures. Thus, [bmPy][BF_4_] shows a total decomposition in one step at 440 °C, where a sharp exothermic peak on the DTA curves resulted from the self-combustion effect. The thermal behavior of the confined [bmPy][BF_4_] is different and is associated with the early, and often a gradual or multi-step decomposition of IL in a confined geometry. The mass loss in the confined IL shows an early onset, which was confirmed by others studies also, studying an IL with an imidazolium core [12]. The mass loss at around 150 °C in silica xerogels has earlier been reported to be associated with the loss of bound water and the rest of the mass loss was above this temperature indicating total decomposition of the IL [12]. Also, from geometry considerations, the alkyl chains are more susceptible of breaking away compared with the ring, which is hinged more strongly into the silica structure [12]. Comparatively, thermogravimetric studies by Karout et al. showed that around of 6% of the introduced IL can remain entrapped into the silica matrix [59,60], estimated from the difference in mass loss amount and the amount of IL in the sample. In the case of aerogels synthesized with [bmPy][BF_4_] the first moderate mass loss is observed at higher temperature between 350 and 450 °C (the authors assumed that it can be attributed to adsorbed ionic liquid) and the total IL mass loss comleted at 500 °C. This observation is consistent with adsorption of residual traces of the [bmPy][BF_4_] on the gel network, in place of residual free water and methanol [60].

In the case of an inert atmosphere, only one mass loss occurred between 225 and 450 **°**C (Figure 6a), and similar results were obtained by Wu et al. [7,54] for silica matrix with [bmIm][BF_4_] ionic liquid. Pure [bmIm][BF_4_] displayed the thermal decomposition temperature of around 412 °C and no residue remained after heating above 482 °C [7,54]. The vast majority of the ILs has a high thermal stability usually up to 400 °C [61]. Fox et al. pointed out that nucleophilic anions reduce the thermal stability compared to bulky fluoride containing anions and the alkyl chain length does not have a large effect on the thermal stability (there is no significant correlation in the thermal stabilities of the synthesized compounds with respect to the alkyl chain length) [61]. Kosmulski et al. indicated that thermal stability of an IL depends on the anion type and the effect of the cation is insignificant and, the thermal stability increases with increasing anion size and decreases with its hydrophilicity [62]. The decomposition temperatures depend primarily on the nucleophilicity of the anion, and ILs containing weakly nucleophilic anions have greater thermal stability. Song et al. demonstrated that the imidazolium ILs start to lose mass around 150 °C, while the pyrrolidinium analogs at around 200 °C [63]. Another study demostrated that the decomposition of tetrafluoroborates starts in the temperature range 194–283 °C (with ammonium cations) and occurs typically in 2–4 steps [64]. For [bmIm][BF_4_], the mass loss onset temperature is 424 °C.

Usually, the true stability is a lower temperature than that one provided by a TGA scan [65]. The wet ionic liquids have a mass loss at about 100 °C due to water evaporation, while the remaining IL decomposes as if it was a pure melt salt [64]. Also water contamination of the tetrafluoroborate ionic liquids would decrease the thermal stability of the anion, since BF_4_^-^ is thermodynamically unstable towards hydrolysis to boric acid and HF [51]. In another study, no decomposition in neat pyrrolidinium ionic liquid [bmPy][Tf2N] was seen until at least 400 °C [66]. It is important to remember that TGA only measures mass loss, and since significant chemistry can occur before this, the onset of melting temperatures are often inaccurate in describing thermal stability [66]. Ngo et al. investigated the properties of a series of imidazolium ILs and pointed out that larger and more asymmetrical cations cause lower melting points [67]. Alkyl chain lengthening may cause the disruption of the symmetry and could also lead to lower melting points. By increasing the alkyl chain length, the melting points of imidazolium iodide salts changed from 196 °C to 106 °C and generally decreased, by increasing the alkyl chain length [68]. Results of another study suggest that melting point decreases with increasing alkyl-chain length and larger anion size, and under nitrogen atmosphere, almost no mass loss occurs below 400 °C, and the thermal stability of the ILs is mostly anion dependent [69].

One of the common methods for confining liquids and gases in mesopores is by using a porous matrix made by the sol–gel technique which has given a new class of materials, which consist of an IL in porous oxide matrice, called ionogel [2]. The ionogels are different from normal xerogels. In the latter, the liquid from the sol after gellification is removed during the drying process while in ionogels the dopant IL remains entrapped in the pores of the gelled matrix [12]. The possible conformations of the confined IL were studied by Singh and coworkers using theoretical Hartree–Fock (HF) calculations. When the butyl-methyl-imidazolium cation is confined to a nanopore, the surface oxygen atom of the SiO_2_ matrix interacts with the cation, namely with the C–H groups of imidazolium [12]. In aerogels and xerogels, the ILs bulky cations act also as spacers, leading to increased average pore radius at low IL content, in comparison with similar gels made without IL [2].

Previous studies have shown that short chain ionic liquids can self-aggregate in aqueous solvents [70,71,72]. Alkyl tails of the ionic liquid cations can aggregate through van der Waals attraction resulting in micelle formation. The size of micelles increases with increasing of ionic liquid concentration [71]. It may be supposed that the condensing silica oligomers adsorb onto the micelles, reducing intermicellar repulsion and resulting in aggregation to form initial nuclei. From this point forward, the growth occurs in a cooperative manner, with condensing silica filling the gaps between further aggregating micelles [3]. The probable mechanism of the influence of the ionic liquid in the medium can be explain by the fact that this short-chain IL does not form proper micelles, but it does agglomerate or self-associate, thus providing confinement and promoting the agglomeration of the condensed silica species. The influence of IL on the primary particle size can be attributed to the slowing down the poly-condensation reactions in the presence of IL co-solvent, leading to formation of larger particles. In details, during condensation of the silica oligomers, they adsorb to the polar groups of the IL aggregates, similarly to the condensation of silica in the presence of long alkyl chain cationic surfactants. The self-association of the IL molecules, induced by their hydrophobic aromatic and alkyl groups, brings about the cooperative character of the condensation of the silica, resulting in larger primary silica particles [4].

## 5. Conclusions

New composite materials have been prepared using a short alkyl chain ionic liquid, N-butyl-3-methylpyridinium tetrafluoroborate ([bmPy][BF_4_]) as templating agent, and tetramethoxysilan (TMOS) and methyl-trimethoxysilane (MTMS) as silica precursors. The amount of IL was varied over a broad range, and the structural and thermophysical properties of the composites have been analyzed by IR, SEM, SANS and thermogravimetry. The scattering measurements revealed a characteristic hierarchical structure of the prepared composites, in which the silica grain size doubles with the increase of the IL content. The thermal behaviour of the composites was studied in air and nitrogen atmospheres. In nitrogen, the IL decomposition starts above 150 °C, and completes mainly in the 150–460 °C temperature interval. Above 460 °C, a slight mass loss occurs by the slow partial oxidation of the remaining carbon, as indicated by the black color of the solid residues. The decomposition progressed differently in the synthetic air atmosphere. There, IL decomposition and oxidation took place in the 170–430 °C temperature interval, in several distinct steps. A two-step mass loss was observed in the IL containing xerogels, accompanied by three overlapped exothermic peaks on the heat flow curves. This retarding effect could be attributed to the intermediate decomposition products forming inside the silica matrix pores. The mass spectrometric evolved gas analysis showed that the degradation of the longer butyl side chain of the cation starts at lower temperature (above 150 °C) through a C-N bond cleavage, initiated by the nucleophilic attack of a fluorine ion. From the ionic liquid geometry, it is clear that the longer alkyl chain is likely to be more susceptible to breaking away followed by the other molecular fragments and this would cause the mass loss of IL in the confined geometry to take place in more than one step. The addition of the short-chain IL to the reaction mixture leads to the slowing of the condensation reactions. This results in gradual morphology change in the nanometer size range appearing as the growth of the primary silica particles with increasing amount of the IL. The morphology change of the micrometer sized particles can be noticed in the sample with the maximum amount of ionic liquid, the particle surfaces become rougher. Shrinkage and syneresis are reduced during gelation and aging, therefore due to a limited collapse of the pores during solvent extraction, highly porous silica matrixes can be synthesized. Our further studies will concern preparation of IL templated mesoporous matrixes with ionic liquid extraction by using solvent mixtures or by high temperature thermal treatments.

## Figures and Tables

**Table 1:** Sample compositions.

Sample	IL/Si Molar Ratio	IL [g]	TMOS [g]	MTMS [g]
BF4-0	0	0	4.95	0.55
BF4-0.3	0.3	2.60	4.95	0.55
BF4-0.4	0.4	3.46	4.95	0.55
BF4-0.5	0.5	4.33	4.95	0.55
BF4-0.6	0.6	5.20	4.95	0.55

**Table 2:** FT-IR bands assignments of the xerogels and of the thermally treated samples.

Band Positions		Assignments
BF4-0.3, BF4-0.6 (Xerogels)	BF4-0.3, BF4-0.6 (Heated Samples)	
3479 cm^−1^, 3553 cm^−1^		O-H stretching bands of hydrogen-bonded water molecules and SiO-H stretching of surface silanols hydrogen-bonded to molecular water (SiO-H…H_2_O) [35].
3194–3081 cm^−1^	Changes with thermal treatment. Intense in xerogels and decreases in the heated samples.	OH stretching vibrations [36].
2965–2977 cm^−1^ and 2878–2879 cm^−1^	Disappear after thermal treatment.	Stretching vibrations of alkyl chains [41].
2324–1981 cm^−1^	2324–1981 cm^−1^	Vibrations of organic residue, molecular water and silica network [38].
1636–1637 cm^−1^	-	The adsorbed water deformation vibration [35,36,37,38].
1635 cm^−1^	1635 cm^−1^	Vibrations of SiO_2_ network and molecular water [38].
1277–1279 cm^−^^1^	Disappear after thermal treatment.	Symmetric CH_3_ bending band from Si–CH_3_ [37,42].
1057–1024 cm^−1^	Shifted to higher wave numbers after calcination, due to elimination of the IL and strengthening of the silica matrix [26].	Si-O-Si asymmetric stretching vibrations [35].
1507 cm^−1^ and 1468 cm^−1^	Shifted to 1383 cm^−1^.	Pyridinium group vibrations [46].The band at 1383 cm^−1^ is present also in the bulk IL and is generally assigned to stretching vibrations of C-H bond [41] and C-C bond [47]. This shows that some IL still remains entrapped in the silica matrix, after the one hour thermal treatment.
1468 cm^−1^	Disappear after thermal treatment.	C-H stretching vibrations of the alkyl chain [14].
800 cm^−1^	800 cm^−1^	Symmetric stretching vibrations of Si-O-Si [35,40].
765 cm^−1^	-	Characteristic band of BF_4_ group [43] (overlaps with the symmetric stretching vibrations of Si-O-Si appearing at 800 cm^−1^ and 680 cm^−1^).
687 cm^−1^	Almost disappears since the CH_3_ groups from the silica matrix were also eliminated.	Symmetric stretching vibration of the C-Si bond (reported at 676 cm^−1^ in [45]).
680 cm^−1^	680 cm^−1^	Symmetric stretching vibrations of Si-O-Si [35,40].

**Table 3:** Textural parameters obtained from nitrogen porosity measurements.

Sample	Specific Surface Area [m^2^/g]	Mean Pore Width [nm]	Total Pore Volume [cm^3^/g]
BF4-0	206	2.4	1.02 × 10^−1^
BF4-0.3	11.3	6.1	2.2 × 10^−2^
BF4-0.4	4.1	7.6	5.0 × 10^−3^
BF4-0.5	3.2	5.3	4.5 × 10^−3^
BF4-0.6	1.1	2.1	6.9 ×10^−3^

## Data Availability

Experimental data are available upon request from the authors.

## Supplementary Materials

The following are available online at https://www.mdpi.com/article/10.3390/ma14174918/s1, Figure S1: EDS spectra of sample BF4-0 (a) and BF4-4 (b) taken at different locations, with linear, Figure S2: Representative SEM images of IL-silica composites. The scale bar corresponds to 30 μm, Figure S3: Ion current curves of the main fragments evaporated during the heat treatment of sample, Figure S4: Ion current curves of the main fragments characteristic to the pyridyl group during the heat treatment of sample BF4-0.4, Figure S5: Ion current curves of the main fragments characteristic to the hydrocarbon side chains during the heat treatment of sample BF4-0.4, Figure S6: Analog mass spectra of the volatiles evolved at 335.4 °C during heating the sample BF4-0.4, Figure S7: Analog mass spectra of the volatiles evolved at 415 °C during heating the sample BF4-0.4, Table S1: Atomic compositions of ionogels measured by EDS. Standard deviations are calculated from averaging the results of 4–7 spectra taken at different points, Table S2: Characteristic temperature ranges and thermal parameters for heating in synthetic air and inert atmosphere.

## References

[1] Zhou Y. (2005). Recent advances in ionic liquids for synthesis of inorganic nanomaterials. Curr. Nanosci..

[2] Karout A., Pierre A.C. (2007). Silica xerogels and aerogels synthesized with ionic liquids. J. Non-Cryst. Solids.

[3] Putz A.M., Len A., Ianăşi C., Savii C., Almásy L. (2016). Ultrasonic preparation of mesoporous silica using pyridinium ionic liquid. Korean J. Chem. Eng..

[4] Almásy L., Putz A.-M., Len A., Plestil J., Savii C. (2017). Small-angle scattering investigation of silica xerogels and sonogels prepared with ionic liquid pyridinium tetrafluoroborate. Process. Appl. Ceram..

[5] Viau L., Néouze M.-A., Biolley C., Volland S., Brevet D., Gaveau P., Dieudonné P., Galarneanu A., Vioux A. (2012). Ionic liquid mediated sol-gel synthesis in the presence of water or formic acid: Which synthesis for which material?. Chem. Mater..

[6] Zhou Y., Schattka J.H., Antonietti M. (2004). Room-temperature ionic liquids as template to monolithic mesoporous silica with worm-like pores via a sol-gel nanocasting technique. Nano Lett..

[7] Wu C.M., Lin S.Y., Chen H.L. (2012). Structure of a monolithic silica aerogel prepared from a short-chain ionic liquid. Micropor. Mesopor. Mater..

[8] Wu C.M., Lin S.Y., Kao K.Y., Chen H.L. (2014). Self-organization of a hydrophilic short-chain ionic liquid confined within a hydrophobic nanopore. J. Phys. Chem. C.

[9] Zhou Y., Antonietti M. (2003). Preparation of highly ordered monolithic super-microporous lamellar silica with a room-temperature ionic liquid as template via the nanocasting technique. Adv. Mater..

[10] Zhang H., Liu S. (2019). Preparation of ordered mesoporous silica materials templated by ionic liquids in alkaline condition. J. Porous Mater..

[11] Göbel R., Hesemann P., Weber J., Möller E., Friedrich A., Beuermann S., Taubert A. (2009). Surprisingly high, bulk liquid-like mobility of silica-confined ionic liquids. Phys. Chem. Chem. Phys..

[12] Singh M.P., Singh R.K., Chandra S. (2010). Thermal stability of ionic liquid in confined geometry. J. Phys. D Appl. Phys..

[13] Göbel R., Friedrich A., Taubert A. (2010). Tuning the phase behavior of ionic liquids in organically functionalized silica ionogels. Dalton Trans..

[14] Reinert L., Batouche K., Lévêque J.-M., Muller F., Bény J.-M., Kebabi B., Duclaux L. (2012). Adsorption of imidazolium and pyridinium ionic liquids onto montmorillonite: Characterisation and thermodynamic calculations. Chem. Eng. J..

[15] Askalany A., Olkis C., Bramanti E., Lapshin D., Calabrese L., Proverbio E., Freni A., Santori G. (2019). Silica-supported ionic liquids for heat-powered sorption desalination. ACS Appl. Mater. Interfaces.

[16] Trif L., Franguelli F.P., Lendvay G., Majzik E., Béres K., Bereczki L., Szilágyi I.M., Pawar R.P., Kótai L. (2021). Thermal analysis of solvatomorphic decakis (dimethylammonium) dihydrogendodecatungstate hydrates. J. Therm. Anal. Calorim..

[17] Zaharescu M., Predoana L., Pandele-Cusu J., Klein L., Aparicio M., Pandele-Cusu J. (2018). Thermal Analysis on Gels, Glasses, and Powders. Handbook of Sol-Gel Science and Technology.

[18] Cao Y., Mu T. (2014). Comprehensive investigation on the thermal stability of 66 ionic liquids by thermogravimetric analysis. Ind. Eng. Chem. Res..

[19] Hao Y., Peng J., Hu S., Li J., Zhai M. (2010). Thermal decomposition of allyl-imidazolium-based ionic liquid studied by TGA–MS analysis and DFT calculations. Thermochim. Acta.

[20] Clough M.T., Geyer K., Hunt P.A., Mertes J., Welton T. (2013). Thermal decomposition ofcarboxylate ionic liquids: Trends and mechanisms. Phys. Chem. Chem. Phys..

[21] Dou Q., Liu L., Yang B., Lang J., Yan X. (2017). Silica-grafted ionic liquids for revealing the respective charging behaviors of cations and anions in supercapacitors. Nat. Comm..

[22] Han L., Park S.-W., Park D.-W. (2009). Silica grafted imidazolium-based ionic liquids: Efficient heterogeneous catalysts for chemical fixation of CO_2_ to a cyclic carbonate. Energy Environ. Sci..

[23] Siewniak A., Forajter A., Szymańska K. (2020). Mesoporous silica-supported ionic liquids as catalysts for styrene carbonate synthesis from CO_2_. Catalysts.

[24] Shi T., Livi S., Duchet J., Gérard J.-F. (2020). Ionic liquids-containing silica microcapsules: A potential tunable platform for shaping-up epoxy-based composite materials?. Nanomaterials.

[25] Mohamedali M., Ibrahim H., Henni A. (2020). Imidazolium based ionic liquids confined into mesoporous silica MCM-41 and SBA-15 for carbon dioxide capture. Micropor. Mesopor. Mater..

[26] Shi F., Zhang Q., Li D., Deng Y. (2005). Silica-gel-confined ionic liquids: A new attempt for the development of supported nanoliquid catalysis. Chem. Eur. J..

[27] Qian C., Yao C., Yang L., Yang B., Liu S., Liu Z. (2020). Preparation and application of silica films supported imidazolium-based ionic liquid as efficient and recyclable catalysts for benzoin condensations. Catal. Lett..

[28] Vioux A., Viau L., Volland S., Le Bideou J. (2010). Use of ionic liquids in sol-gel; ionogels and applications. Comptes Rendus Chim..

[29] Le Bideau J., Viau L., Vioux A. (2011). Ionogels, ionic liquid based hybrid materials. Chem. Soc. Rev..

[30] Hesemann P., Viau L., Vioux A., Levy D., Zayat M. (2015). Silica Ionogels and Ionosilicas. The Sol-Gel Handbook.

[31] Andrzejewska E., Marcinkowska A., Zgrzeba A. (2017). Ionogels—Materials containing immobilized ionic liquids. Polimery.

[32] Guo L.Y., Shi J., He J.H., Huang J.Y., Huang P.C. (2015). Synthesis and characterization of supported on silica based ionic liquids. Appl. Mech. Mater..

[33] Chen X., Put B., Sagara A., Gandrud K., Murata M., Steele J.A., Yabe H., Hantschel T., Roeffaers M., Tomiyama M. (2020). Silica gel solid nanocomposite electrolytes with interfacial conductivity promotion exceeding the bulk Li-ion conductivity of the ionic liquid electrolyte filler. Sci. Adv..

[34] Almásy L. (2021). New measurement control software on the Yellow Submarine SANS instrument at the Budapest Neutron Centre. J. Surf. Investig. X-ray Synchrotron Neutron Tech..

[35] Al-Oweini R., El-Rassy H. (2009). Synthesis and characterization by FTIR spectroscopy of silica aerogels prepared using several Si(OR)_4_ and R”Si(OR’)_3_ precursors. J. Mol. Struct..

[36] Innocenzi P. (2003). Infrared spectroscopy of sol–gel derived silica-based films: A spectra-microstructure overview. J. Non-Cryst. Solids.

[37] Socrates G., Socrates G. (2004). Infrared and Raman Characteristic Group Frequencies: Tables and Charts.

[38] Lenza R.F.S., Vasconcelos W.L. (2001). Preparation of silica by sol-gel method using formamide. Mater. Res..

[39] Icopini G.A., Brantley S.L., Heaney P.J. (2005). Kinetics of silica oligomerization and nanocolloid formation as a function of pH and ionic strength at 25 °C. Geochim. Cosmochim. Acta.

[40] Gopal N.O., Narasimhulu K.V., Rao J.L. (2004). EPR, Optical, infrared and Raman spectral studies of actinolite mineral. Spectrochim. Acta Part A Mol. Biomol. Spectrosc..

[41] Gunzler H., Gremlich H.U. (2004). IR Spectroscopy: An Introduction.

[42] Stuart B.H., Ando D.J. (2005). Infrared Spectroscopy: Fundamentals and Applications, Book Series: Analytical Techniques in the Sciences.

[43] Shi F., Deng Y. (2005). Abnormal FT-IR and FTRaman spectra of ionic liquids confined in nano-porous silica gel. Spectrochim. Acta Part A Mol. Biomol. Spectrosc..

[44] Warring S.L., Beattie D.A., McQuillan A.J. (2016). Surficial siloxane-to-silanol interconversion during room-temperature hydration/dehydration of amorphous silica films observed by ATR-IR and TIR-Raman spectroscopy. Langmuir.

[45] Jitianu A., Crisan M., Meghea A., Rau I., Zaharescu M. (2002). Influence of the silica based matrix on the formation of iron oxide nanoparticles in the Fe_2_O_3_–SiO_2_ system, obtained by sol–gel method. J. Mater. Chem..

[46] Jin Y., Wang P., Yin D., Liu J., Qin L., Yu N., Xie G., Li B. (2007). Gold nanoparticles prepared by sonochemical method in thiol-functionalized ionic liquid. Colloids Surf. A Physicochem. Eng. Asp..

[47] Damlin P., Kvarnström C., Ivaska A. (2004). Electrochemical synthesis and in situ spectroelectrochemical characterization of poly(3,4-ethylenedioxythiophene) (PEDOT) in room temperature ionic liquids. J. Electroanal. Chem..

[48] Gubanova N.N., Baranchikov A.Y., Kopitsa G.P., Almásy L., Angelov B., Yapryntsev A.D., Rosta L., Ivanov V.K. (2015). Combined SANS and SAXS study of the action of ultrasound on the structure of amorphous zirconia gels. Ultrason. Sonochem..

[49] Tomchuk O.V., Avdeev M.V., Ivankov O.I., Bulavin L.A., Aksenov V.L. (2019). Features of colloidal aggregation in tetraethoxysilane-water-ethanol ternary mixtures by small-angle neutron scattering. J. Surf. Investig. X-Ray Synchrotron Neutron Tech..

[50] Tomchuk O.V., Bulavin L.A., Pipich V., Ryukhtin V., Ivankov O.I., Aksenov V.L., Avdeev M.V. (2020). Fractal aggregation in silica sols in basic tetraethoxysilane/ethanol/water solutions by small-angle neutron scattering. J. Mol. Liq..

[51] Fagadar-Cosma E., Dudás Z., Birdeanu M., Almásy L. (2014). Hybrid organic — silica nanomaterials based on novel A_3_B mixed substituted porphyrin. Mater. Chem. Phys..

[52] Lázár I., Forgács A., Horváth A., Király G., Nagy G., Len A., Dudás Z., Papp V., Balogh Z., Moldován K. (2020). Mechanism of hydration of biocompatible silica-casein aerogels probed by NMR and SANS reveal backbone rigidity. Appl. Surf. Sci..

[53] Margaca F.M.A., Miranda Salvado I.M., Teixeira J. (1999). Small angle neutron scattering study of silica gels: Influence of pH. J. Non-Cryst. Solids.

[54] Wu C.-M., Lin S.-Y. (2012). Preparation and fractal-structure characterization of monolithic silica aerogel with a short-chain Ionic liquid as the solvent. Trans. Mater. Res. Soc. Jpn..

[55] Dudás Z., Fagadar-Cosma E., Len A., Románszki L., Almásy L., Vlad-Oros B., Dascalu D., Krajnc A., Kriechbaum M., Kuncser A. (2018). Improved optical and morphological properties of vinyl-substituted hybrid silica materials incorporating a Zn-metalloporphyrin. Materials.

[56] Becauge G. (1996). Small-angle scattering from polymeric mass fractals of arbitrary mass-fractal dimension. J. Appl. Cryst..

[57] Chal B., Roiban L., Masenelli-Varlot K., Baeza G.P., Yrieix B., Foray G. (2021). 3D multi-scale quantification of industrially relevant ultra-porous silicas by low-dose electron tomography combined with SANS. J. Non-Cryst. Solids.

[58] Teixeira J. (1988). Small-angle scattering by fractal systems. J. Appl. Cryst..

[59] Karout A., Pierre A.C. (2009). Porous texture of silica aerogels made with ionic liquids as gelation catalysts. J. Sol-Gel Sci. Technol..

[60] Karout A., Pierre A.C. (2009). Silica gelation catalysis by ionic liquids. Catal. Commun..

[61] Fox D.M., Awad W.H., Gilman J.W., Maupin P.H., De Long H.C., Trulove P.C. (2003). Flammability, thermal stability, and phase change characteristics of several trialkylimidazolium salts. Green Chem..

[62] Kosmulski M., Gustafsson J., Rosenholm J.B. (2004). Thermal stability of low temperature ionic liquids revisited. Thermochim. Acta.

[63] Song Y., Liu L., Zhu X., Wang X., Jia H., Xiao X., Yu H., Yang X. (2008). Physicochemical properties of ionic liquids based on imidazolium/pyrrolidinium cations and maleate/phthalate anions. Solid State Ion..

[64] Kärnä M., Lahtinen M., Valkonen J. (2009). Preparation and characterization of new low melting ammonium-based ionic liquids with ether functionality. J. Mol. Struct..

[65] Valkenburg M.E.V., Vaughn R.L., Williams M., Wilkes J.S. (2005). Thermochemistry of ionic liquid heat-transfer fluids. Thermochim. Acta.

[66] Del Sesto R.E., McCleskey T.M., Macomber C., Ott K.C., Koppisch A.T., Baker G.A., Burrell A.K. (2009). Limited thermal stability of imidazolium and pyrrolidinium ionic liquids. Thermochim. Acta.

[67] Ngo H., LeCompte K., Hargens L., McEwen A. (2000). Thermal properties of imidazolium ionic liquids. Thermochim. Acta.

[68] Ozdemir S., Varlikli C., Oner I., Ocakoglu K., Icli S. (2010). The synthesis of 1,8-naphthalimide groups containing imidazolium salts/ionic liquids using I^−^, PF_6_^−^, TFSI^−^ anions and their photophysical, electrochemical and thermal properties. Dyes Pigm..

[69] Hsieh Y.N., Kuei C.H., Chou Y.K., Liu C.C., Leu K.L., Yang T.H., Wang M.Y., Ho W.Y. (2010). Facile synthesis of polymerized ionic liquids with high thermal stability. Tetrahedron Lett..

[70] Almásy L., Turmine M., Perera A. (2008). Structure of aqueous solutions of ionic liquid 1-butyl-3-methylimidazolium tetrafluoroborate by small-angle neutron scattering. J. Phys. Chem. B.

[71] Bowers J., Butts C.P., Martin P.J., Vergara-Gutierrez M.C., Heenan R.K. (2004). Aggregation behavior of aqueous solutions of ionic liquids. Langmuir.

[72] Sastry N.V., Vaghela N.M., Macwan P.M., Soni S.S., Aswal V.K., Gibaud A. (2012). Aggregation behavior of pyridinium based ionic liquids in water—surface tension, ^1^H NMR chemical shifts, SANS and SAXS measurements. J. Colloid Interface Sci..

