# Supplementary material for: Butyl-Methyl-Pyridinium Tetrafluoroborate Confined in Mesoporous Silica Xerogels: Thermal Behaviour and Matrix-Template Interaction
Source: Materials (Basel). 2021 Aug 29;14(17):4918. doi: 10.3390/ma14174918 (PMC8433830; doi:10.3390/ma14174918)
Supplement: Supplementary file 1 [file materials-14-04918-s001.zip › materials-1303886-SI.pdf]

materials

MDPI

Supplementary Materials

Butyl-methyl-pyridinium Tetrafluoroborate Confined in Mesoporous Silica Xerogels: Thermal Behaviour and Matrix-template Interaction

Ana-Maria Putz, László Almásy, Zsolt Endre Horváth and László Trif

1. EDS Spectra of IL-silica Composites

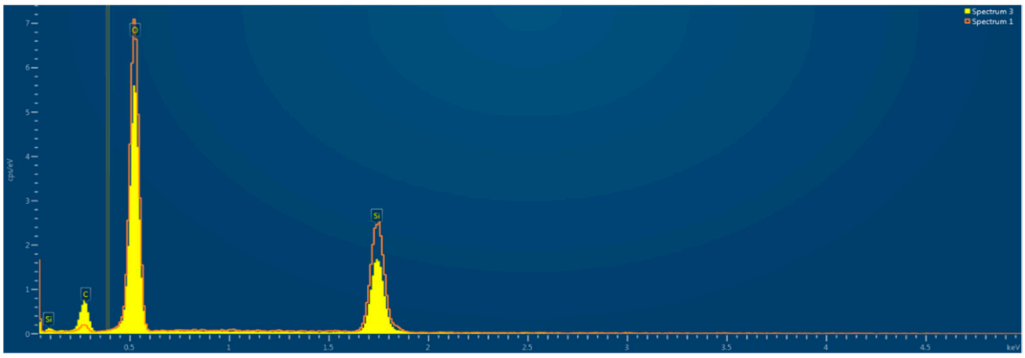

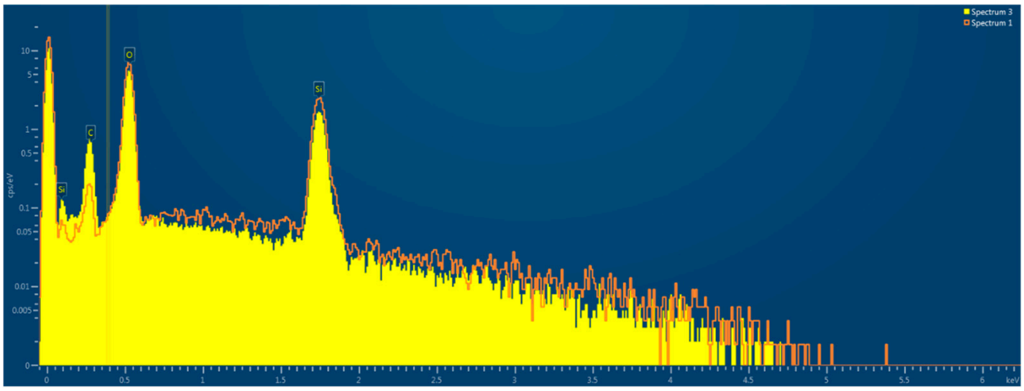

(a)

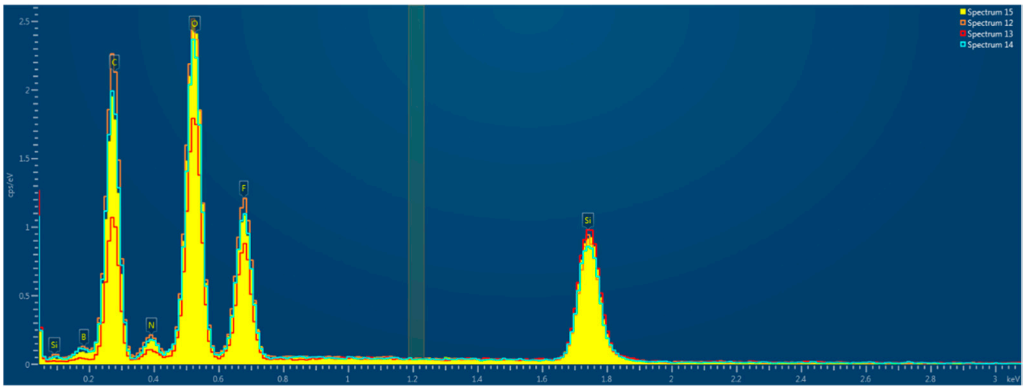

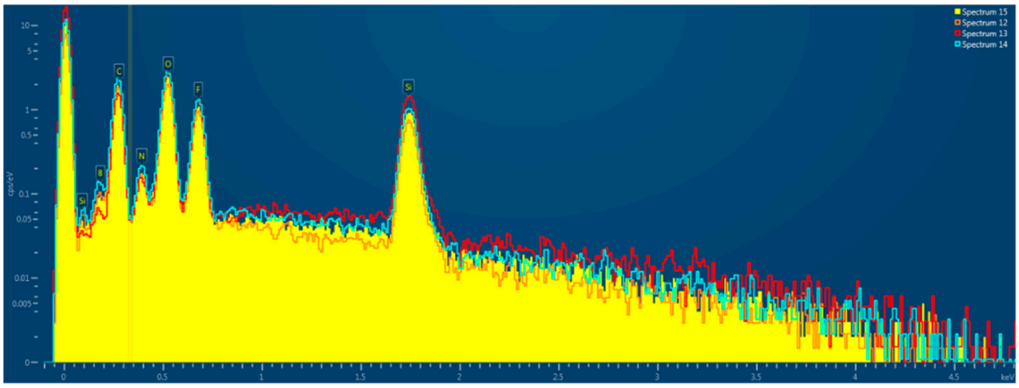

(**b**)

Figure S1. EDS spectra of sample BF4-0 (**a**) and BF4-4 (**b**) taken at different locations, with linear and logarithmic intensity scales.

Table S1. Atomic compositions of ionogels measured by EDS. Standard deviations are calculated from averaging the results of 4–7 spectra taken at different points.

Sample	B [%]	C [%]	N [%]	O [%]	F [%]	Si [%]
BF4-0		9.4 ± 8.4		60.5 ± 3.4		30.0 ± 5.0
BF4-0.3	6.1 ± 1.1	4.1 ± 1.5	3.4 ± 0.1	26.4 ± 0.8	11.5 ± 0.6	12.5 ± 1.3
BF4-0.4	5.7 ± 0.4	42.3 ± 1.1	3.8 ± 0.2	23.8 ± 0.3	13.2 ± 0.2	11.2 ± 0.7
BF4-0.5	6.4 ± 0.3	48.7 ± 0.3	4.3 ± 0.2	17.1 ± 0.4	15.2 ± 0.5	8.2 ± 0.2
BF4-0.6	6.6 ± 0.4	53.9 ± 1.6	4.7 ± 0.3	12.3 ± 1.0	16.2 ± 0.4	6.3 ± 1.1

2. SEM Images of IL-silica Composites

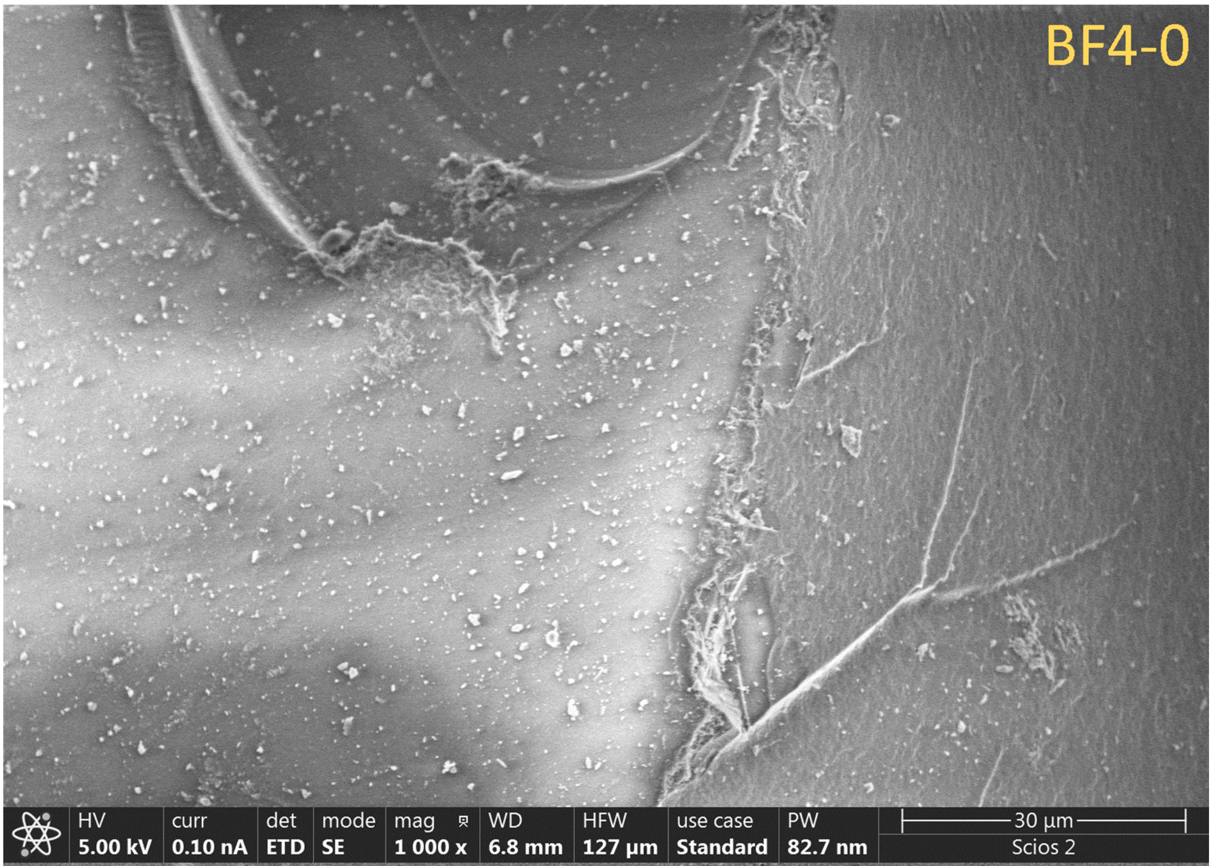

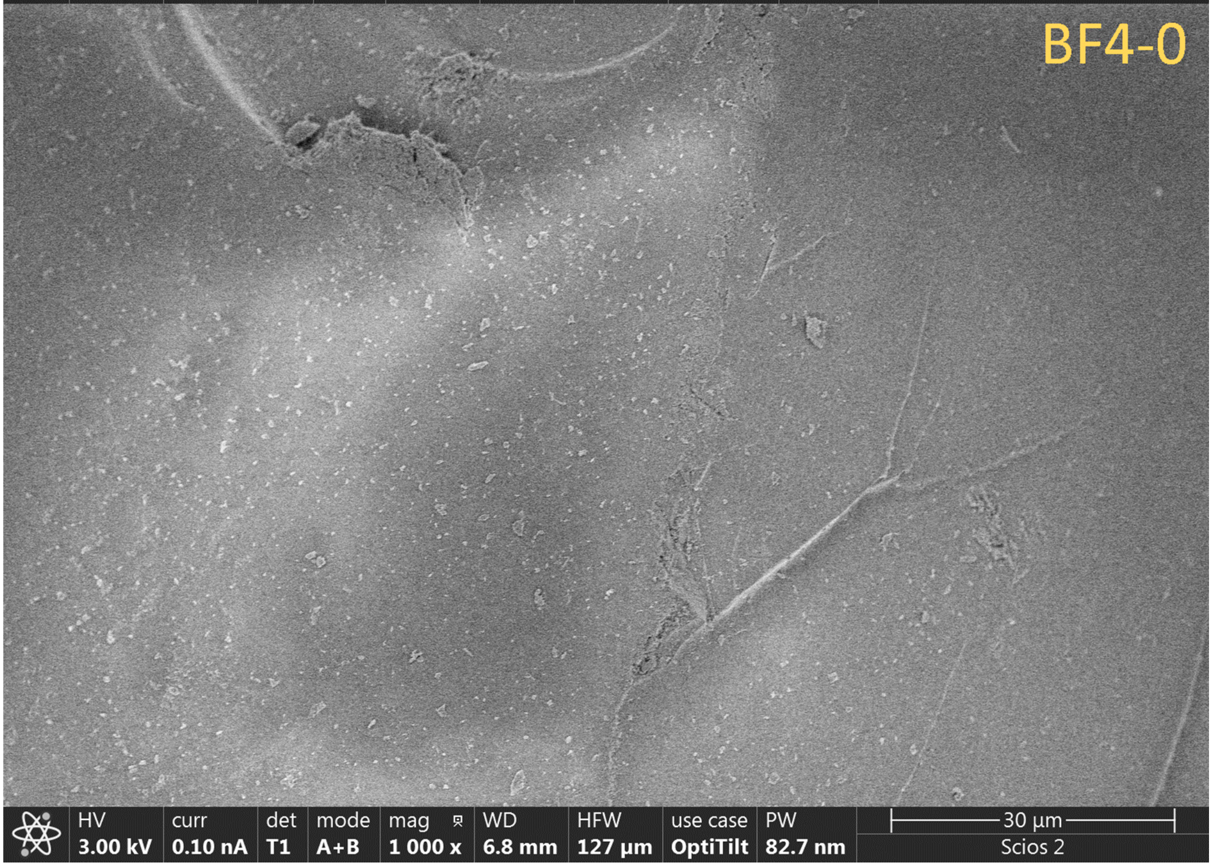

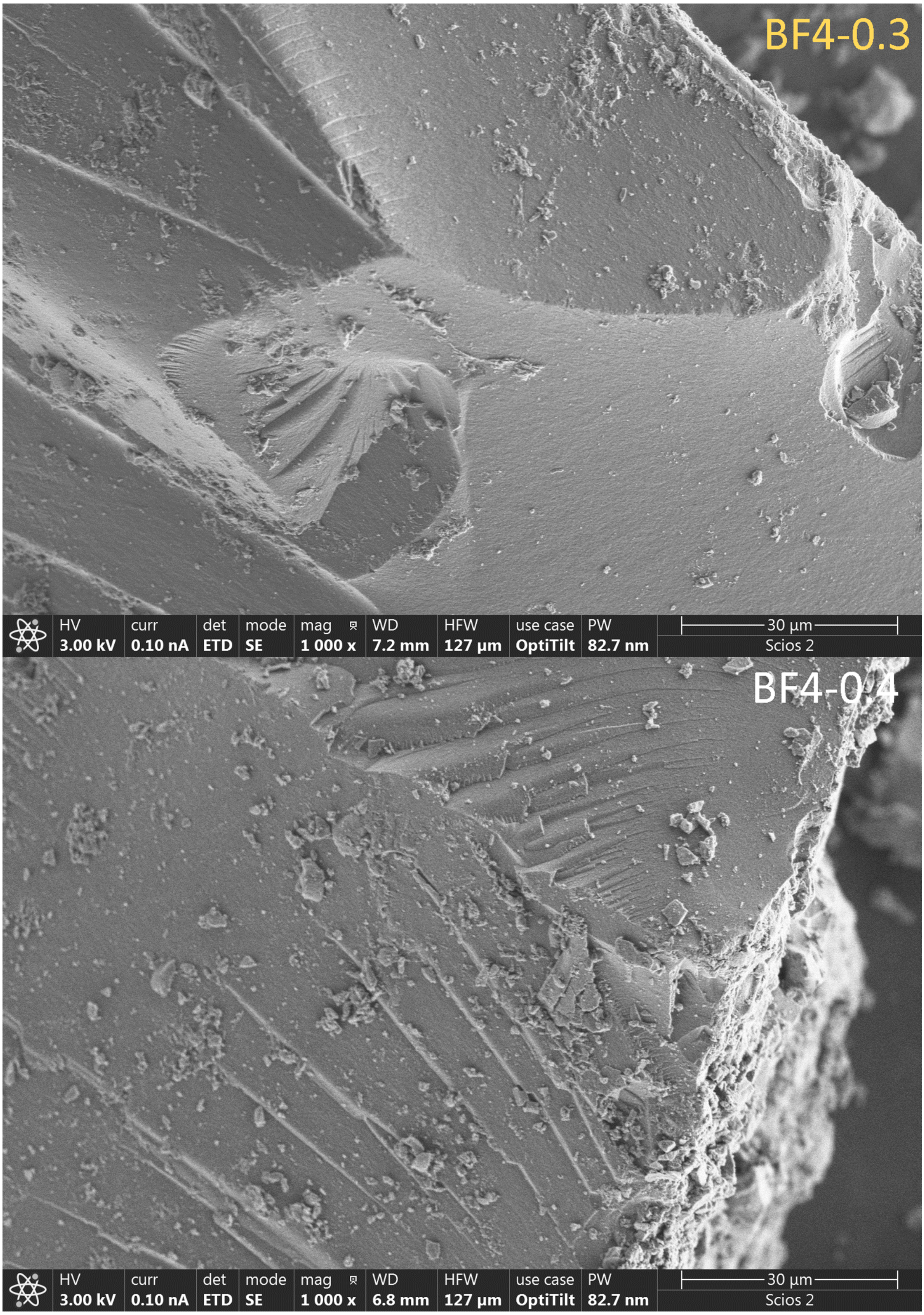
BF4-0.3
HV 3.00 kV
curr 0.10 nA
det ETD
mode SE
mag 1 000 x
WD 7.2 mm
HFW 127 µm
use case OptiTilt
PW 82.7 nm
30 µm
Scios 2
BF4-0.4
HV 3.00 kV
curr 0.10 nA
det ETD
mode SE
mag 1 000 x
WD 6.8 mm
HFW 127 µm
use case OptiTilt
PW 82.7 nm
30 µm
Scios 2

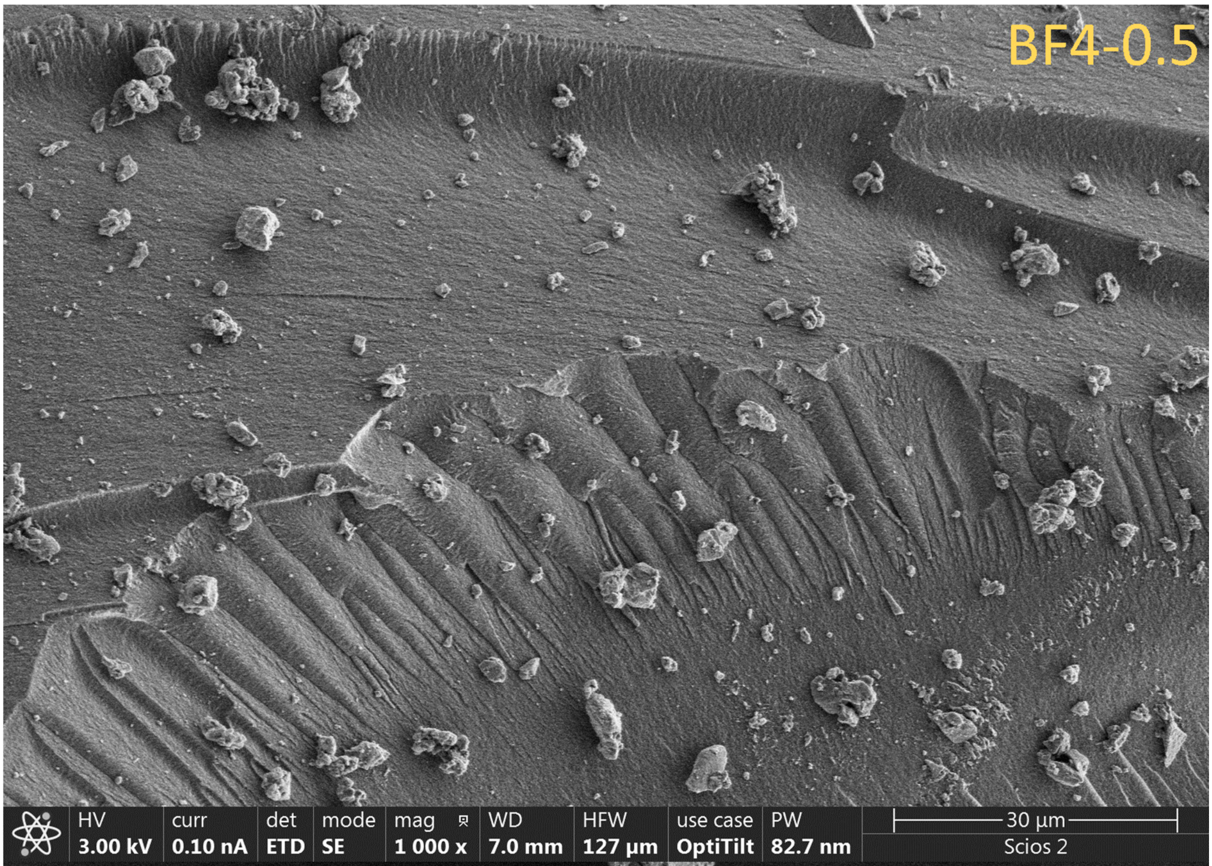
BF4-0.5
HV 3.00 kV
curr 0.10 nA
det ETD
mode SE
mag 1 000 x
WD 7.0 mm
HFW 127 µm
use case OptiTilt
PW 82.7 nm
30 µm
Scios 2

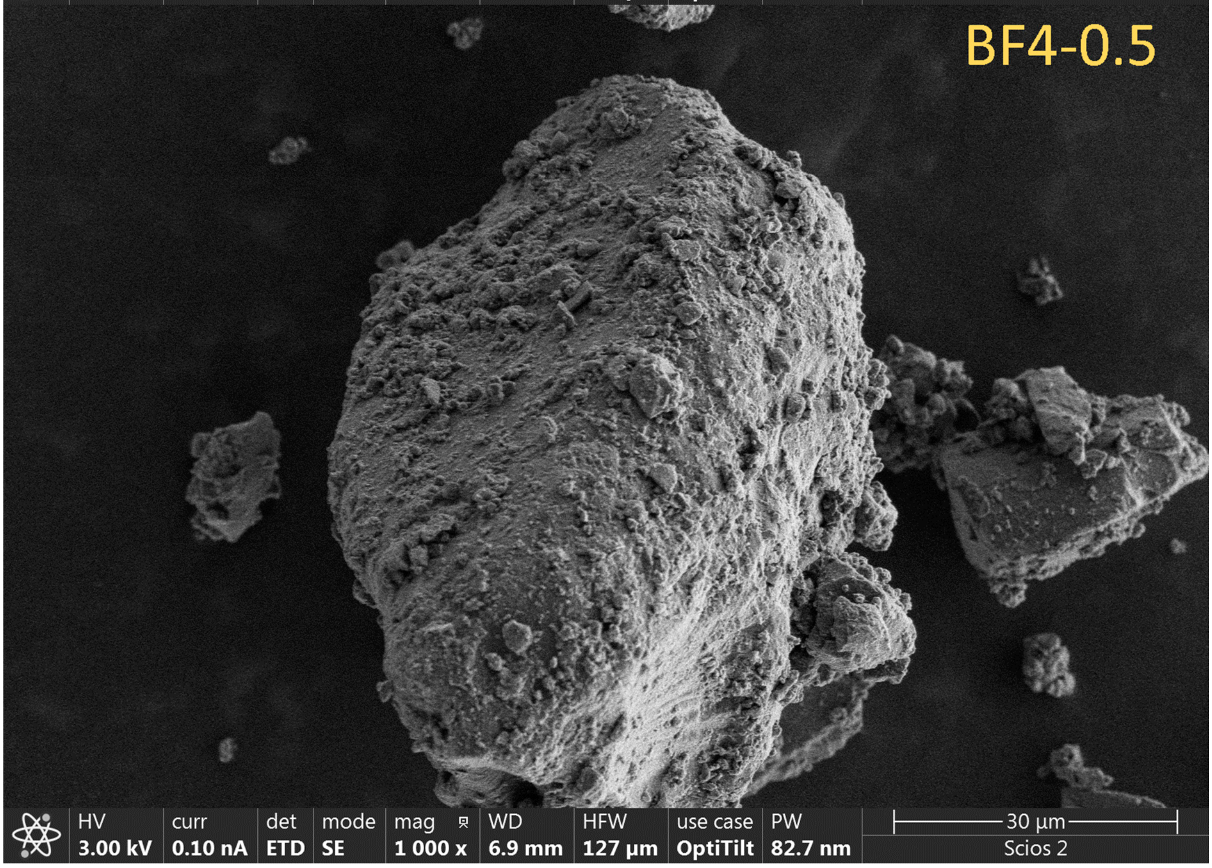
BF4-0.5
HV 3.00 kV
curr 0.10 nA
det ETD
mode SE
mag 1 000 x
WD 6.9 mm
HFW 127 µm
use case OptiTilt
PW 82.7 nm
30 µm
Scios 2

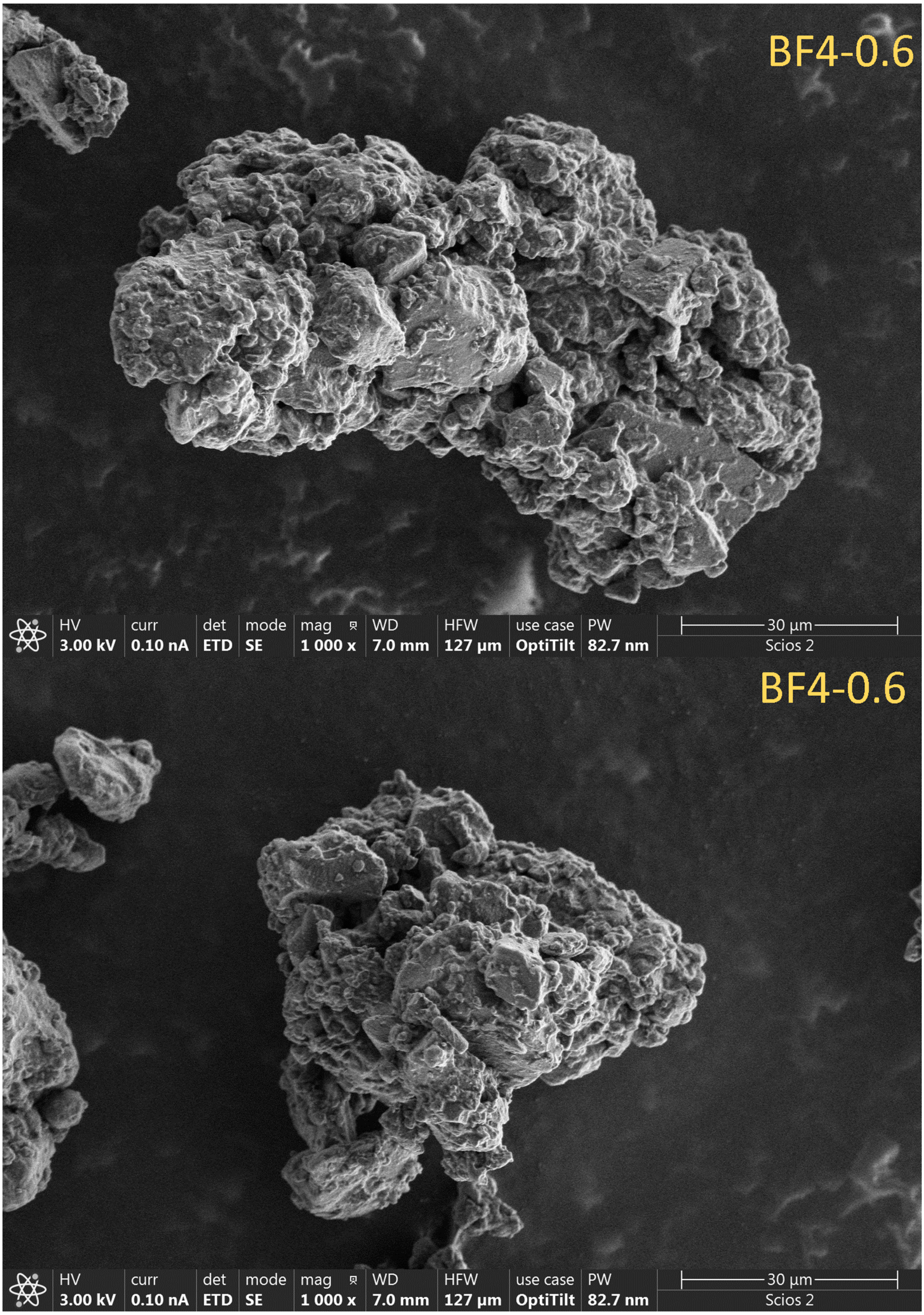
BF4-0.6
HV 3.00 kV
curr 0.10 nA
det ETD
mode SE
mag 1 000 x
WD 7.0 mm
HFW 127 µm
use case OptiTilt
PW 82.7 nm
30 µm
Scios 2
BF4-0.6
HV 3.00 kV
curr 0.10 nA
det ETD
mode SE
mag 1 000 x
WD 7.0 mm
HFW 127 µm
use case OptiTilt
PW 82.7 nm
30 µm
Scios 2

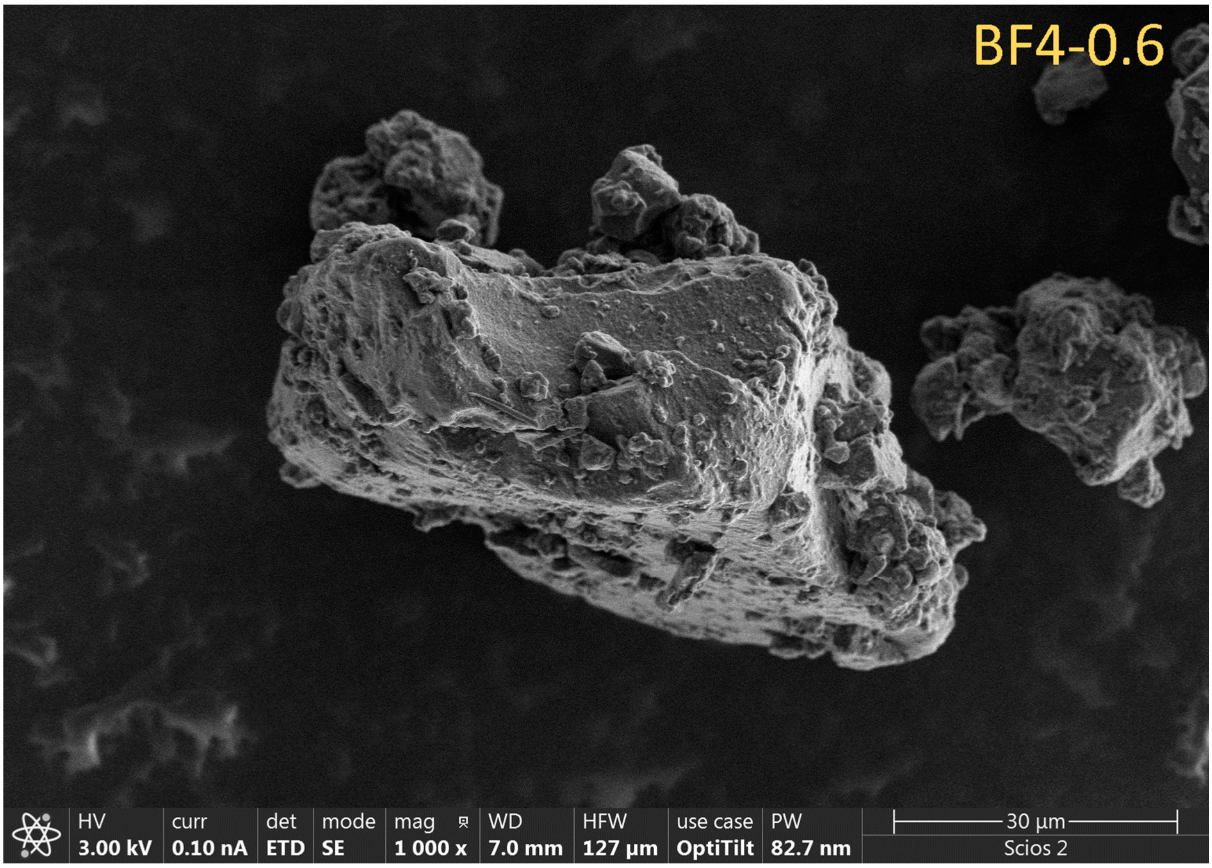

Figure S2. Representative SEM images of IL-silica composites. The scale bar corresponds to 30 µm.

3. Characteristics of Thermal Analysis and Mass Spectroscopy Instruments

3.1 *TA*

Weighing range: +/- 1000 mg (in large range); +/- 200 mg (in small range)
Weighing precision: +/- 0.005 %
TG resolution: 0.2 µg (in large range) and 0.02 µg (in small range)
Baseline dynamic drift (one hour): 10 µg
TG reproducibility up to 1200 °C: <50 µg
DSC rod – Resolution: 10 µW (with the S-type DSC rod used)

3.2 *MSEGA*

Detector: C-SEM/Faraday
Min. detection limit, C-SEM: <1 ppm
Min. detection limit, Faraday: <20 ppm
Resolution, settable at 10 % peak height, amu: 0.5–2.5
Contribution to neighboring mass: 40 to 41 <10 ppm

4. Thermal Analysis Results

Table S2. Characteristic temperature ranges and thermal parameters for heating in synthetic air and inert atmosphere.

Step	Temperature Range (°C)	Δ*m* (%)	DTG Peak Temperature (°C)	DSC Peak Temperature (°C)	DSC Onset Temperature (°C)	Δ*H* (kJ mol⁻¹)
				Nitrogen		
BF4-0						
1	34.2–170	−8	107.4	108.9	61.5	230.23
2	170–460.3	−1.38	280.7	-	-	-
3	460.3–689.4	−0.45	-	-	-	-
Total mass loss (30–700 °C)				**−9.83%**		
BF4-0.3						
1	35.2–170	−0.36	-	-	-	-
2	170–460	−57.76	411.5	411.2	338.4	464.96
3	460–693.5	−0.79	-	-	-	-
Total mass loss (30–700 °C)				**−58.91%**		
BF4-0.4						
1	34.6–170	−0.48	-	-	-	-
2	170–460	−63.96	415.2	413.9	334.9	561
3	460–693.5	−1.02	-	-	-	-
Total mass loss (30–700 °C)				**−65.46%**		
BF4-0.5						
1	33.6–170	−0.41	-	-	-	-
2	170–460	−71.29	417.1	414.8	348	603.84
3	460–693.5	−0.79	-	-	-	-
Total mass loss (30–700 °C)				**−72.49%**		
BF4-0.6						
1	35.6–170	−0.21	-	-	-	-
2	170–460	−83.68	420.7	420.1	357.3	772.96
3	460–693.6	−0.63	-	-	-	-
Total mass loss (30–700 °C)				**−84.52 %**		
				Synthetic Air		
BF4-0						
1	40–170	−8.39	113.9	111.6	63.1	230.93
2	170–430	−2.74	-	-	-	-
3	430–695.7	−2.67	-	A series of overlapping sharp exothermic peaks		-
Total mass loss (30–700 °C)				**−13.8%**		
BF4-0.3						
1	34.5–170	−0.82	-	-	-	-
2	170–430	−46.81	378.9	368.7<br>410.8	288.2	−5619.87
3	430–695.7	−14.4	525	505.7	-	
Total mass loss (30–700 °C)				**−62.03%**		
BF4-0.4						
1	34.5–170	−0.69	-	-	-	-
2	170–430	−52.37	379.6	364.8<br>413.3	288.9	−5858.23
3	430–695.6	−14.8	525.1	497.5	-	

Total mass loss (30–700 °C)				**−67.86%**		
BF4-0.5						
1	34.5–170	−0.59	-	-	-	-
2	170–430	−58.36	378.7	368<br>412.7	288.7	−6042.14
3	430–696	−15.18	525.2	499	-	
Total mass loss (30–700 °C)				**−74.13%**		
BF4-0.6						
1	34.8–170	−0.19	-	-	-	-
2	170–430	−69.49	377.8	372.3<br>419.3	298.7	−6240.38
3	430–695.9	−15.91	524.4	490	-	
Total mass loss (30–700 °C)				**−85.59 %**		

5. Ion Current Curves and Mass Spectra in Evolved Gas Analysis Coupled with Thermal Analysis

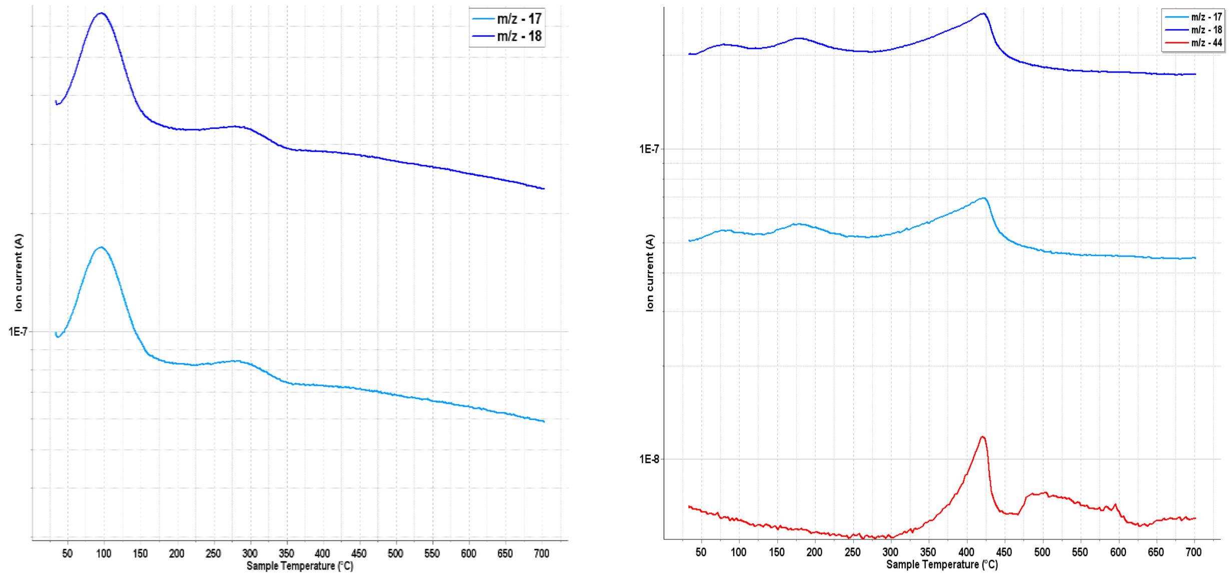

(**a**) (**b**)

Figure S3. Ion current curves of the main fragments evaporated during the heat treatment of sample BF4-0 (**a**) and the main fragments of water and carbon dioxide during the heat treatment of sample BF4-0.4 (**b**).

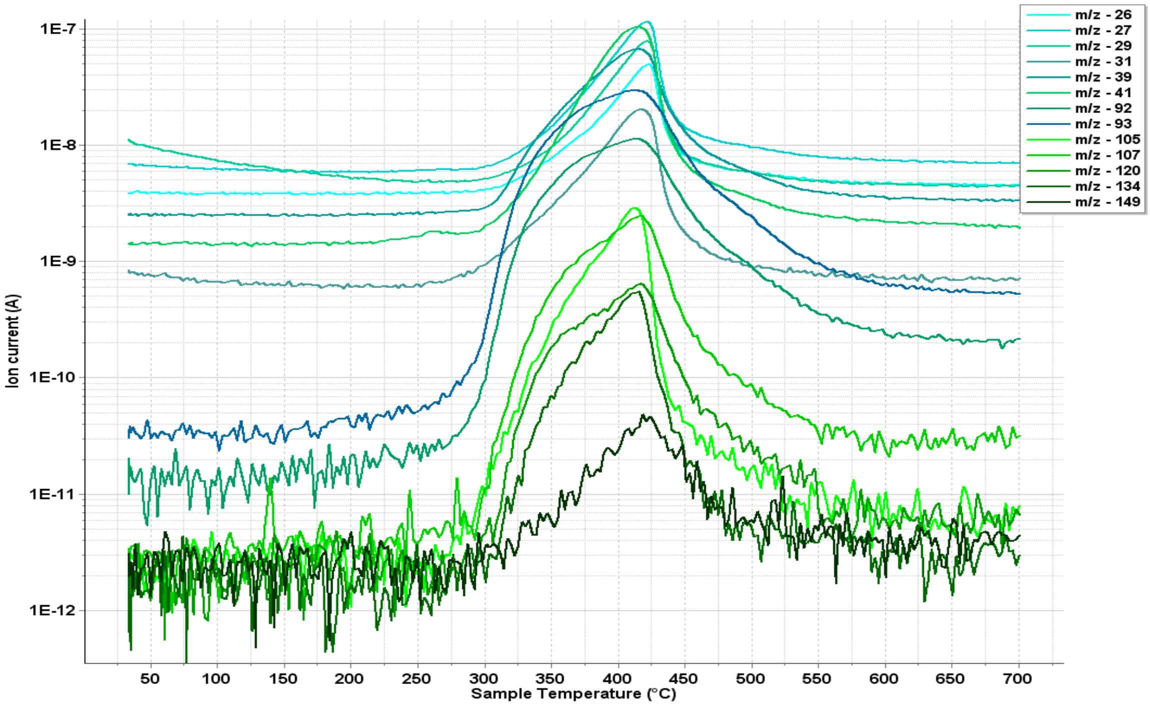

Figure S4. Ion current curves of the main fragments characteristic to the pyridyl group during the heat treatment of sample BF4-0.4.

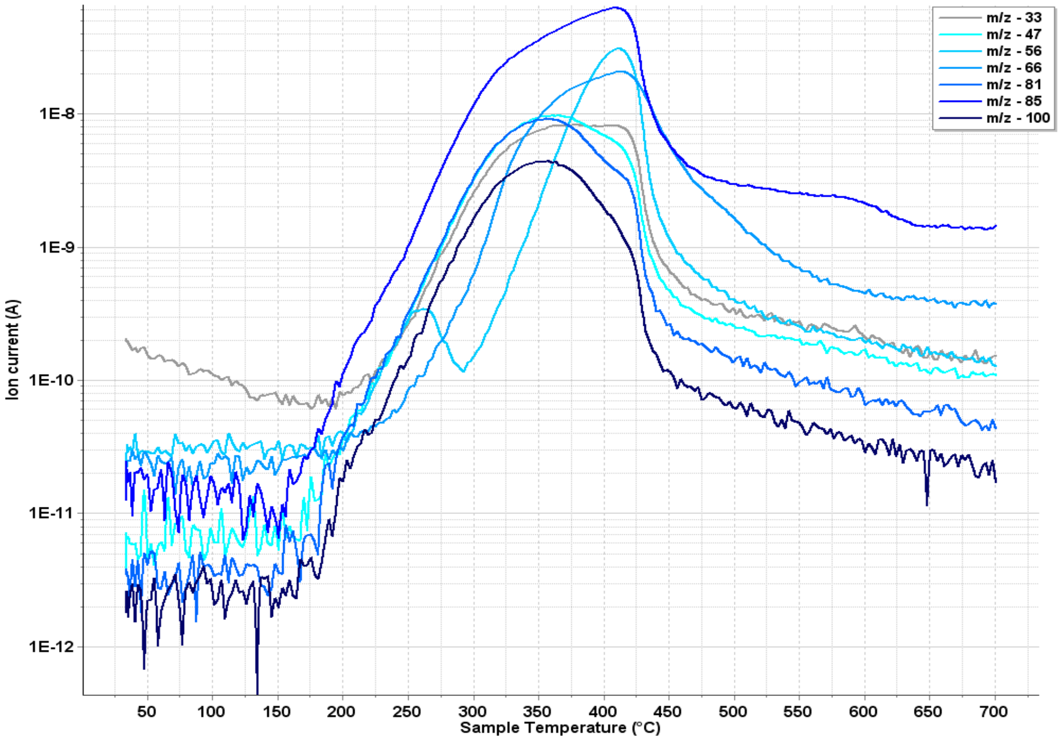

Figure S5. Ion current curves of the main fragments characteristic to the hydrocarbon side chains during Table 4.

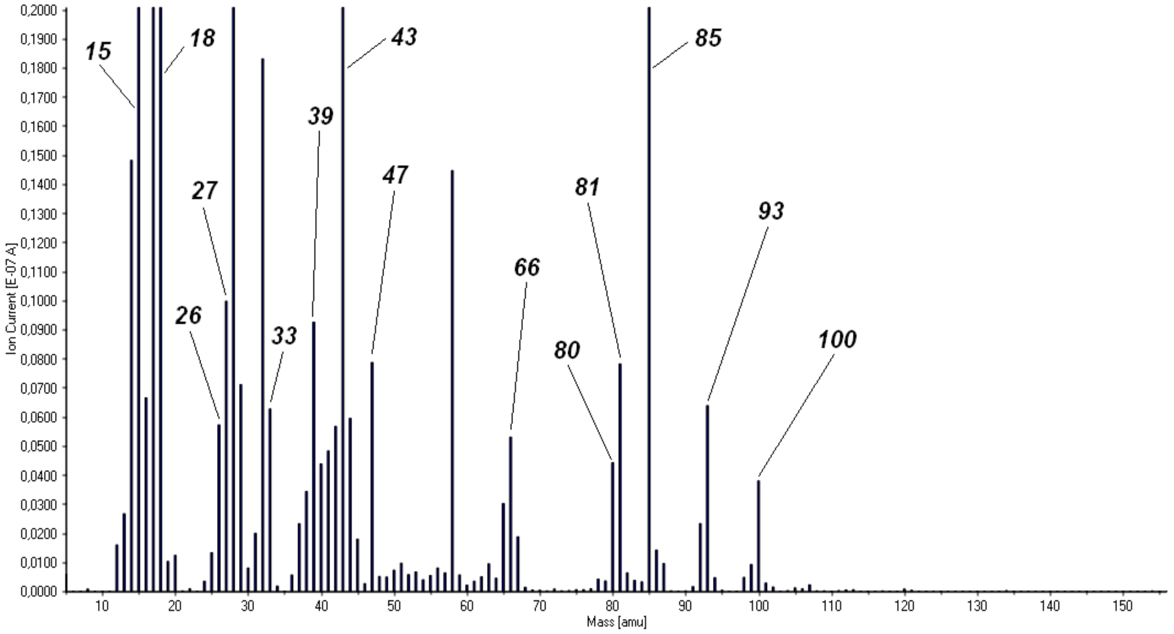

Figure S6. Analog mass spectra of the volatiles evolved at 335.4 °C during heating the sample BF4-0.4.

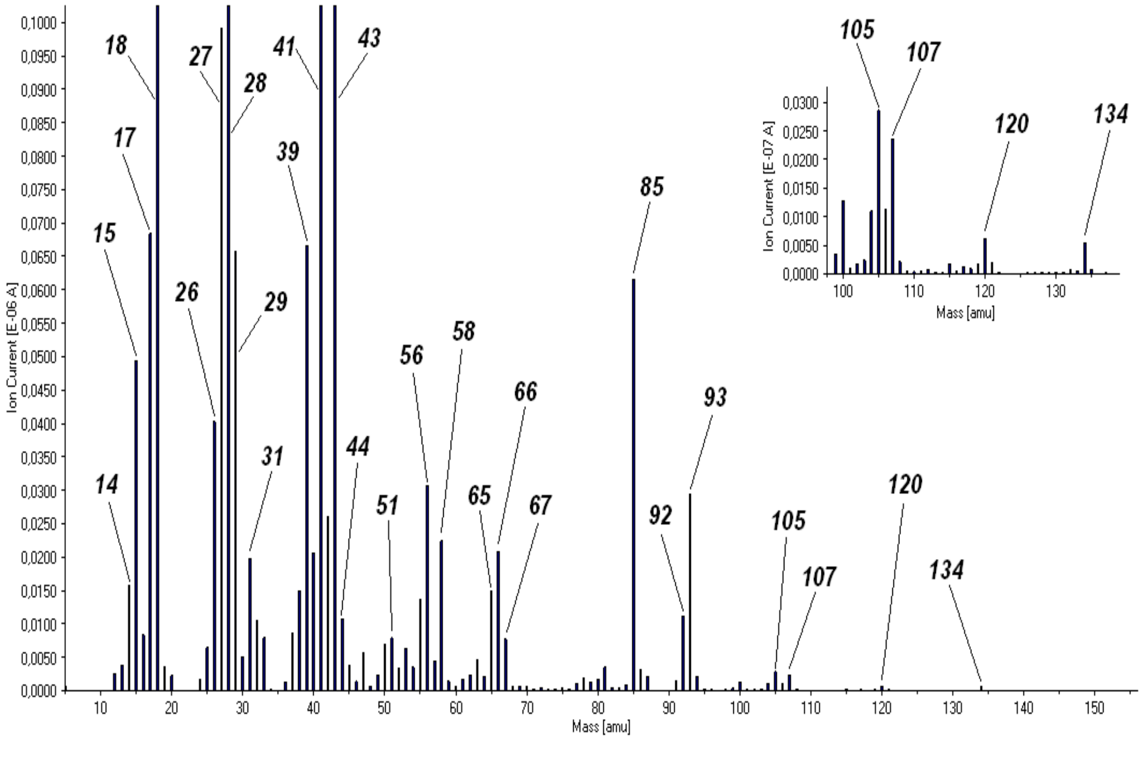

Figure S7. Analog mass spectra of the volatiles evolved at 415 °C during heating the sample BF4-0.4.
